# Acute optogenetic induction of the prodromal endophenotype of CA1 hyperactivity causes schizophrenia-related deficits in cognition and salience attribution

**DOI:** 10.1038/s41537-024-00513-w

**Published:** 2024-10-08

**Authors:** Sampath K. T. Kapanaiah, Christina Grimm, Dennis Kätzel

**Affiliations:** 1 Institute of Applied Physiology, Ulm University, Ulm, Germany; 2 Present Address: Center for Biomedical Imaging (CIBM), Lausanne, Switzerland; 3 Present Address: School of Engineering, Neuro-X Institute, EPFL, Lausanne, Switzerland

**Keywords:** Schizophrenia, Neural circuits

## Abstract

Hyperactivity of the human anterior hippocampus has been reported to spread from its CA1 subfield to the subiculum around the onset of first-episode psychosis and could be a cellular target for early therapeutic intervention in the schizophrenia prodrome. However, to what extent CA1 hyperactivity actually causes schizophrenia-related symptoms remains unknown. Here, we mimic this endophenotype by direct optogenetic activation of excitatory cells in the homologous mouse region, ventral CA1 (vCA1) and assess its consequence in multiple schizophrenia-related behavioural tests. We find that hyperactivity of vCA1 causes hyperlocomotion and impairments of spatial and object-related short-term habituation (spatial novelty-preference and novel-object recognition memory) and spatial working memory, whereas social interaction, spatial exploration, and anxiety remain unaltered. Stimulation of the ventral subiculum, in contrast, only increased locomotion and exploration. In conclusion, CA1 hyperactivity may be a direct driver of prodromal cognitive symptoms and of aberrant salience assignment leading to psychosis.

## Introduction

Hyperactivity of the human hippocampus constitutes a repeatedly documented physiological endophenotype of schizophrenia^1^. It is observed as elevated baseline (BL) activity at rest^2–6^ and during sensory processing^7–10^. Such basal hyperactivity may entail failures to regulate hippocampal activity in dependence on cognitive demands, as for example observable as a reduced activation during memory retrieval^11^ or impaired habituation during repeated exposure to salient stimuli^8,12^. Indeed, elevated hippocampal BL activity in early-psychosis patients is inversely correlated with task-induced activation of the hippocampus^13^. Correspondingly, hippocampal hyperactivity measured as regional cerebral blood volume (CBV) correlates with the severity of psychotic symptoms^3^; and hippocampal hyperactivity measured with resting-state fMRI correlates with increased negative symptom score and reduced cognitive function, specifically in the domains of attention, working memory and visual memory^14^. Mechanistically, as detailed elsewhere^1,15^, these correlations may be related to the central role of the hippocampus for the attribution of appropriate levels of salience and its regulation by short-term habituation, as faster habituation of hippocampal activity to repeatedly presented salient stimuli correlates with relational memory performance in healthy subjects (but not in patients where both processes are impaired)^12^.

Anatomically, such hyperactivity has mainly been localized to the anterior human hippocampus in early psychosis-patients^3,4,13^ although it spreads to its posterior subdivision later^16^. Importantly, within the hippocampal circuit, hyperactivity appears to start in CA1 in the prodromal phase of at-risk individuals in whom schizophrenia has not been diagnosed yet, affects the major CA1-projection target—the subiculum—around the first psychotic episode, and spreads to other hippocampal subfields subsequently^3,4,17^. This spreading hyperactivity is accompanied by early anterior hippocampal atrophy^3,4^, and a smaller hippocampus—the strongest volume loss across all brain regions in schizophrenia^18^—is predictive of transition to schizophrenia in prodromal patients^19^. Likewise, hyperactivity in anterior CA1 is the most predictive physiological activity change for such a transition, across examined brain regions^3,4^. Such spreading hyperactivity and atrophy indicate a degenerative process around the years of first-episode psychosis that strongly suggests a necessity of early intervention in schizophrenia therapy^1,20,21^: given that several cellular alterations inside and—potentially as a result—outside the hippocampus occurring around this critical time may not be revertible anymore in established schizophrenia, therapy would need to start in the prodrome and potentially target hyperactivity of anterior CA1 and hippocampal atrophy.

To justify such a—logistically and ethically challenging—early intervention in prodromal at-risk individuals, however, causality between the to-be-treated hyperactivity in anterior CA1 and actual symptoms or transition risk needs to be established. As described above, only a few studies in humans have shown correlations between hippocampal hyperactivity and severity of positive^3^, negative^14^ or cognitive^12,14^ symptoms, and causality is not a necessary conclusion of such correlations. Rodent studies, in contrast, allow direct or indirect induction or targeted modulation of neural activity in specific hippocampal subdivisions and subfields during behavioural assessment of schizophrenia-related deficits, and can therefore be used to determine causal relations between them. In fact, several developmental^22^, genetic^23^ or pharmacological^4^ schizophrenia-related rodent models display hippocampal hyperactivity, and have provided indirect evidence for the relation between hippocampal hyperactivity and schizophrenia-related deficits^1^. Furthermore, direct electrical or chemical induction of hyperactivity in the rodent homologue of the human anterior hippocampus—the ventral hippocampus (vHC)—or even its main output subfield, the ventral subiculum (vSub), specifically—have established that rodent correlates of the positive symptom domain (novelty- or stimulant-induced hyperlocomotion, pre-pulse inhibition, mesolimbic dopaminergic hyperactivity) are caused by vHC and vSub hyperactivity, as reviewed elsewhere^1^. This supports the notion that hyperactivity of the subiculum that occurs around the onset of the first psychotic episode may be causal for psychotic symptoms.

However, to what extent such hyperactivity may also induce cognitive or negative symptoms of the disease—which present already during the prodrome—remains vastly underexplored^24–27^, and the consequence of hyperactivity of the CA1 region, the physiological hallmark of prodromal patients that later transition to psychosis^4^, is unknown to our knowledge. To address those questions, we targeted optogenetic activation specifically to excitatory projection neurons of either the subiculum (vSub) or the CA1 region (vCA1) of the vHC in mice and assessed the consequences of their selective hyperactivity across a comprehensive battery relevant to the three symptom domains of schizophrenia, with a particular focus on cognition and salience attribution by means of short-term habituation.

## Results

### Optogenetic induction of hyperlocomotion by stimulation of vCA1 and vSub

**Fig. 1:**
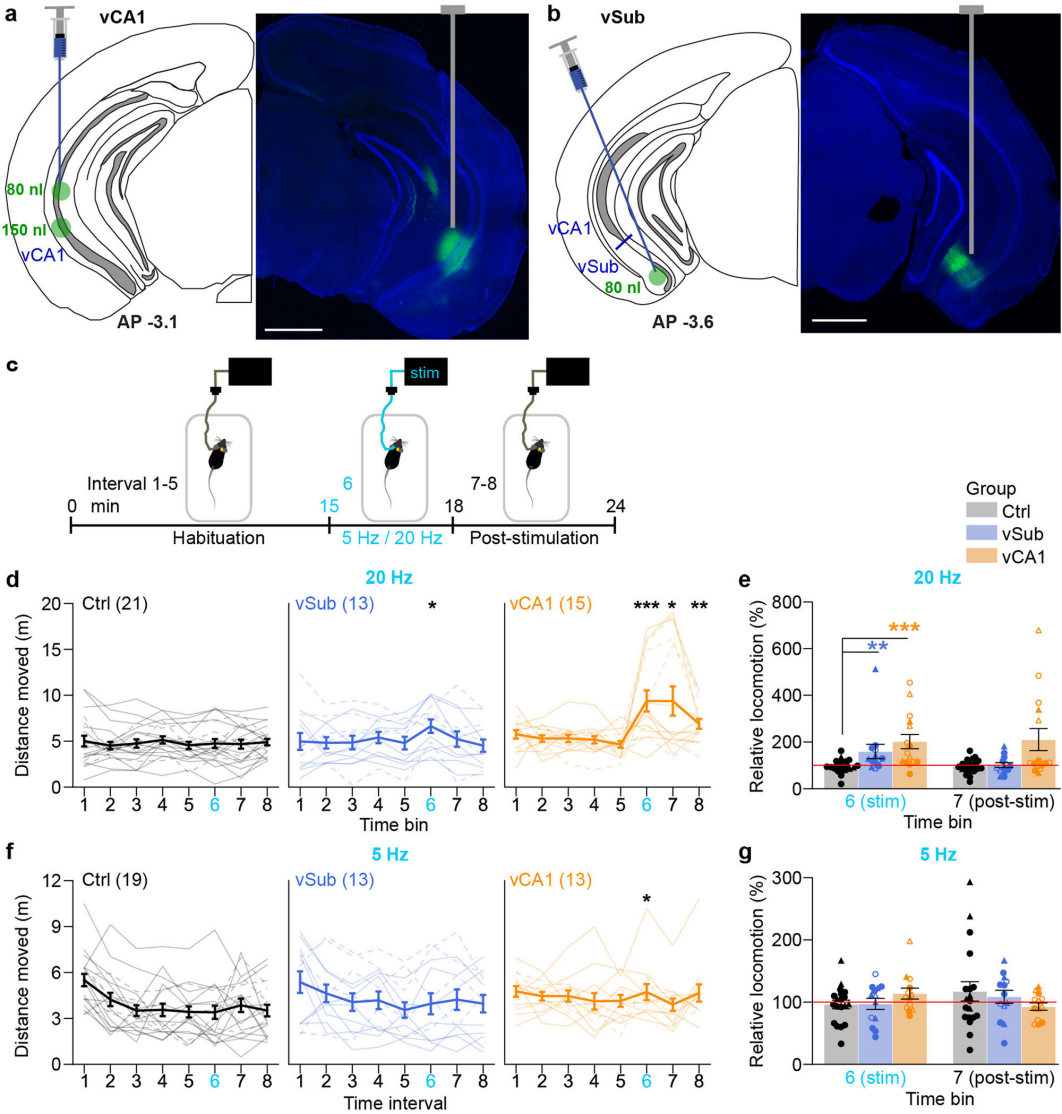
Increase of locomotor activity by optogenetic stimulation of vSub or vCA1. **a**, **b** Illustration (left) and result (right) of the transduction of vCA1 (**a**) and vSub (**b**) regions, respectively. Blue, DAPI; green, native EGFP fluorescence indicating transduced cells; scale, 1 mm. A syringe with a blue needle illustrates the insertion tract for transduction (left); a grey vertical line illustrates the position of the optical fibre (right). **c** Time course of locomotor activity experiments conducted over 24 min (8 intervals) in a novel open field with optogenetic stimulation in 16–18 min (interval 6, cyan). **d**–**g** Locomotor activity expressed as distance moved in 3 min intervals (**d**, **f**) and as relative locomotion during (interval 6) and after (interval 7) optogenetic stimulation divided by the average of the three preceding intervals (3–5; **e**, **g**). Optogenetic stimulation was either conducted at 20 Hz 5 s-on/5 s-off (**d**, **e**) or 5 Hz continuously (**f**, **g**). Individual lines or symbols represent individual subjects, whereby solid lines (**d**, **f**) and circles (**e**, **g**) represent mice where the left hemisphere was stimulated, the remainder represents mice where the right hemisphere was used; open symbols (**e**, **g**) indicate mice with off-target expression in the ventral dentate gyrus (vDG; not shown for Ctrl group); error bars represent s.e.m., asterisks indicate significant difference in non-parametric paired comparisons MWU-test to the control group (Ctrl, black). See Supplementary Table 2 for parametric analysis. *N*-numbers are indicated in brackets for each group (**d**, **f**); two Ctrl and two vCA1 mice did not participate in the 5 Hz experiment. *p < 0.05; **p < 0.01; ***p ≤ 0.001.

To study the causal role of vSub and vCA1 hyperactivity for schizophrenia-related deficits, we transduced either one of these regions in CamKIIα-Cre mice bilaterally with an AAV-vector expressing the optogenetic activator *Chronos* (ChR90)^28^, fused to the green fluorescent protein (GFP), Cre-dependently, yielding the corresponding experimental groups *vSub* (*N* = 13) and *vCA1* (*N* = 15; Fig. 1a, Supplementary Fig. 1, and Supplementary Table 1). About half of the vCA1 mice and a minority of vSub-mice showed additional expression in the *Stratum lacunosum moleculare* of the lateral arm of the ventral dentate gyrus (vDG; Supplementary Figs. 1 and 2 and Supplementary Table 1). Animals transduced with a similar vector which expressed only GFP served as controls, whereby animals receiving vSub vs vCA1 transduction were balanced approximately 1:1 and were combined as a single control group (*Ctrl, N* = 21). Fibre-optic cannulas with 200 μm optical fibres were implanted into the transduced region to deliver blue (473 nm) laser light with an output power of 2.5–5 mW. Previous studies with combined optogenetics and physiological measurements have demonstrated that optogenetic stimulation effectively enhances the firing of hippocampal pyramidal neurons, as for example shown for CA1 neurons of the dorsal hippocampus stimulated with short pulses at 25 Hz^29^ or for several 100 ms^30^. Furthermore, more continuous optogenetic activation of vCA1 cells caused increased local (vHC) multi-unit-activity and BOLD-responses in vCA1, vSub and some of its major projection targets, including entorhinal cortex, striatum and the lateral septum^31,32^.

Excessive novelty- or psychostimulant-induced hyperlocomotion is a well-established rodent correlate of the positive symptom domain^1,33^, and reduced habituation of locomotor activity is also seen in schizophrenia patients^34^. Such hyperlocomotion can be evoked reliably by chemical stimulation of the vHC^35–37^ and also, more specifically, by 20 Hz electrical stimulation of its output stage, the vSub^38^. We have previously shown that 20 Hz optogenetic stimulation of vHC excitatory cells likewise induces hyperlocomotion, whereby this behavioural readout can be used to identify the optimal hemisphere and optical power individually for each animal^26^. In this previous study, we had optically and virally targeted only the vSub, but a majority of experimental animals (16/18) showed expression in adjacent vCA1 in addition to vSub, leaving the specific contribution of each subfield open. In the current study, only vSub-transduced animals void of vCA1 expression, and vice versa, were analysed.

After recovery from surgery, we followed the same approach of using a reproducible increase in locomotor activity to titrate the optical power in the range of 2–5 mW for each animal and hemisphere, as described before in ref. ^26^. We aimed to use the left hemisphere, given that BL hyperactivity in the schizophrenia prodrome is mainly observed on this side^4^, but we also used the right hemisphere when no reproducible locomotor activity increase was found in the tested power range when stimulating the other side (Supplementary Table 1 and Supplementary Fig. 2). Once the optimal hemisphere and power were established, mice were exposed to a novel open field and, after 15 min of habituation, optical stimulation with pulses of 5 ms was unilaterally applied for 3 min—first at 20 Hz and, in a separate experiment, at 5 Hz (Fig. 1c). Whereas intrinsic frequencies occurring during schizophrenia-related hippocampal hyperactivity in humans are not known to our knowledge, we chose 20 Hz as the standard frequency for all subsequent behavioural tests as it is the most well-established frequency for evoking behavioural and physiological rodent correlates of positive symptoms with electrical or optogenetic stimulation of vHC or vSub^1,26,38–41^. We also analysed the effect of 5 Hz, given that theta activity is common in the hippocampus and theta-frequency stimulation of vHC → mPFC afferents could lead to a dopamine-dependent NMDA-receptor LTD in mPFC pyramidal cells, which could, theoretically, cause cognitive deficits^42^.

When analysing total distance moved, both vSub and vCA1 mice displayed higher locomotor activity during 20 Hz stimulation (interval 6) compared to controls (*p* < 0.05, MWU-test; Fig. 1d and Supplementary Table 2 for parametric analysis of this and all other behavioural data); the same was evident when normalizing the movement during interval 6 to the average of the three preceding intervals 3–5 (7–15 min; BL; Fig. 1e). With stimulation of vCA1—but not of vSub—hyperlocomotion was also observed in the intervals after stimulation (Fig. 1d), which may indicate a lasting effect on subjective perception of spatial salience. No such effect was seen with a stimulation frequency of 5 Hz (Fig. 1f, g). This demonstrated that stimulation of vCA1 is sufficient to induce excessive hyperactivity, whereas—as expected^26,38^—this phenotype is also observed with specific stimulation of the vHC output stage, the vSub, alone. Qualitatively, vCA1-mice with off-target expression in vDG appeared to show a stronger hyperlocomotion response than those without vDG-expression (Fig. 1f, g).

### Hyperactivity of the vSub induces a modest increase in exploration

**Fig. 2:**
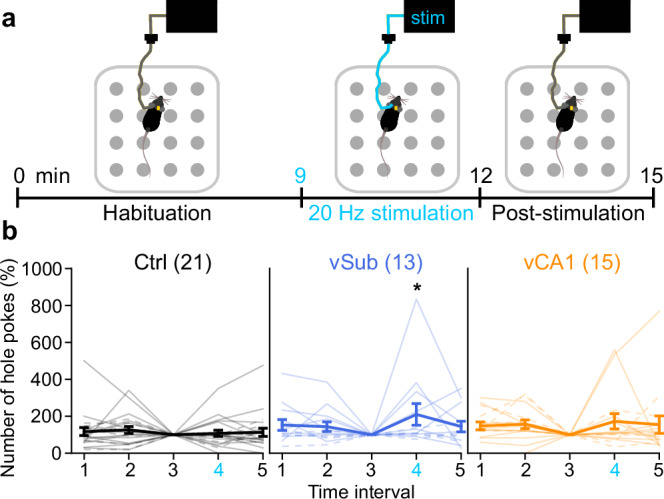
Increase of exploration in the hole-board by vSub stimulation. **a** Timeline of hole-board exploration experiments conducted over 15 min (analysed in five 3 min intervals), with 20 Hz optogenetic stimulation in 10–12 min (interval 4, cyan). **b** Exploration is expressed as the number of hole pokes normalized to the number of pokes made in the third interval (%). Individual lines represent individual subjects, whereas solid and dashed lines represent mice where the left or right, respectively, hemisphere was stimulated; error bars represent s.e.m., asterisk indicates a significant difference in non-parametric paired comparisons (MWU-test) to the control group (Ctrl, black), which is, however, not detected with parametric analysis (*p* = 0.052, one-sided Dunnett’s test, see Supplementary Table 2). *N*-numbers are shown in brackets for each group. *p < 0.05.

Locomotor activity in an open field involves several psychological components, including exploratory drive, locomotor drive, spatial short-term habituation, or anxiety. To investigate the relation between vHC activity and exploration, we assessed the latter by quantifying the number of pokes in 3 min intervals across a 15-min test period on a hole board (Fig. 2a). Optical stimulation of 20 Hz was delivered in the fourth interval whose poke-counts were normalized to that of the preceding interval. Mice stimulated in the vSub showed a modest increase in relative poking (*p* = 0.018) whereas vCA1 activation did not yield a significant increase (*p* = 0.086, MWU-test; Fig. 2b).

### vHC hyperactivity does not affect anxiety levels

**Fig. 3:**
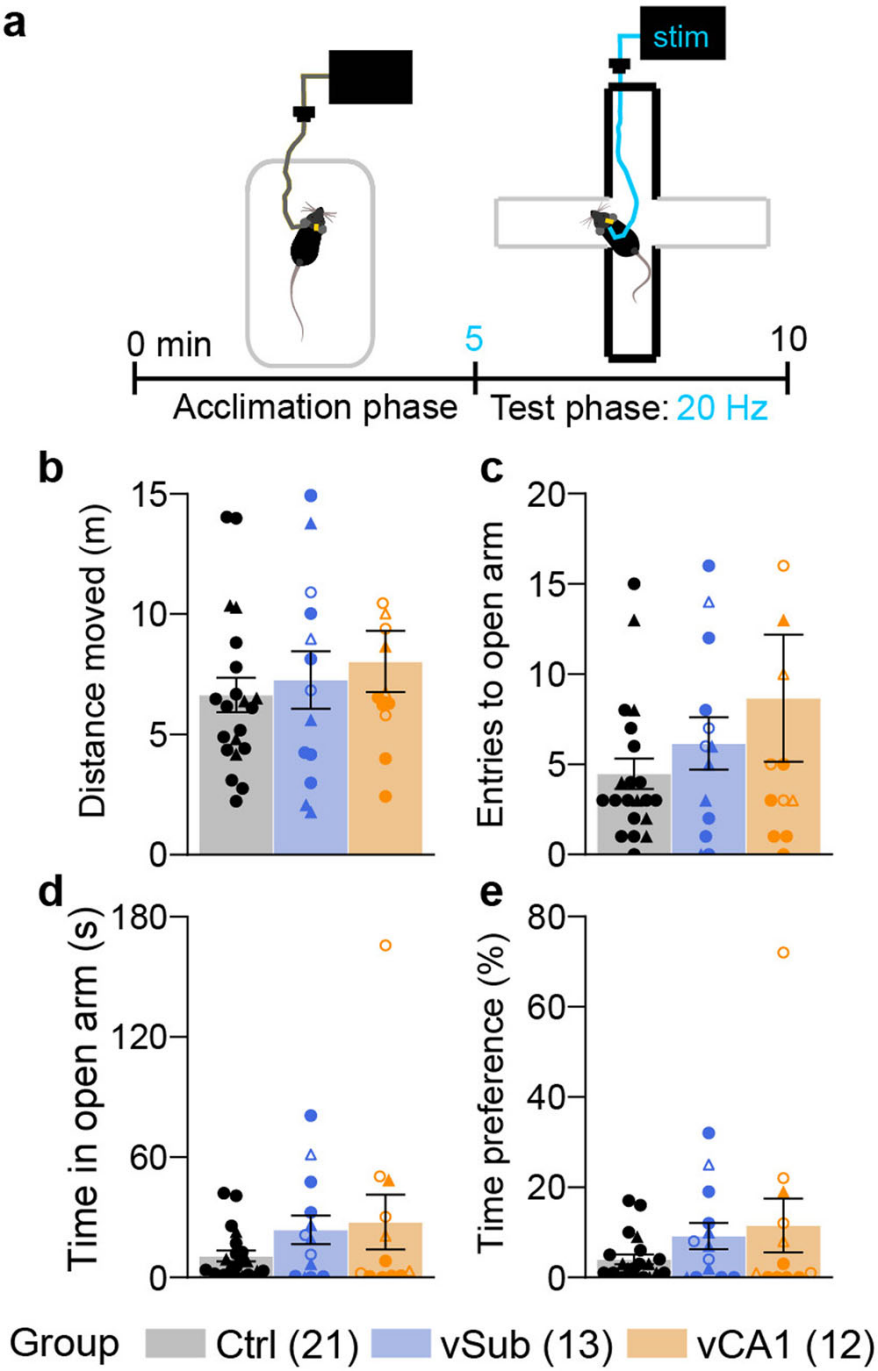
Optogenetic stimulation of vSub or vCA1 does not alter unconditioned anxiety. **a** Time-course of the EPM experiment, consisting of an acclimation phase in a novel open field and a 5-min test phase with concomitant 20 Hz stimulation (5 s-on/5 s-off). **b** Distance moved on the whole EPM during the 5 min. **c**, **d** Number of entries into (**c**) and time spent on (**d**) the open arms. **e** Time spent on the open arms expressed as percentage of time spent on open and closed arms combined (excluding the centre). Individual symbols represent individual subjects, whereby circles and triangles represent mice where the left or the right, respectively, hemisphere was stimulated; open symbols indicate mice with off-target expression in the vDG (not shown for Ctrl group); error bars represent s.e.m., no group differences have been found for any metric (*p* > 0.05; MWU-test comparing each experimental group to the control group (Ctrl, black). See Supplementary Table 2 for parametric analysis. *N*-numbers are shown in brackets for each group; two vCA1 mice were excluded from analysis because they fell off the EPM during testing, and another vCA1 mouse did not participate in this experiment.

To investigate the component of anxiety, we deployed the elevated plus-maze (EPM) test, applying 20 Hz of optical stimulation throughout the 5 min test phase (Fig. 3a). Stimulation of either one of the vHC subregions did not impact the metrics of exploration and anxiety (distance moved, entries to open arm, time in open arm, and time-preference for the open arm) in this test (*p* > 0.2, MWU-test; Fig. 3b–e); in line with our previous study of vHC stimulation^26^.

### vCA1 hyperactivity impairs spatial short-term habituation

**Fig. 4:**
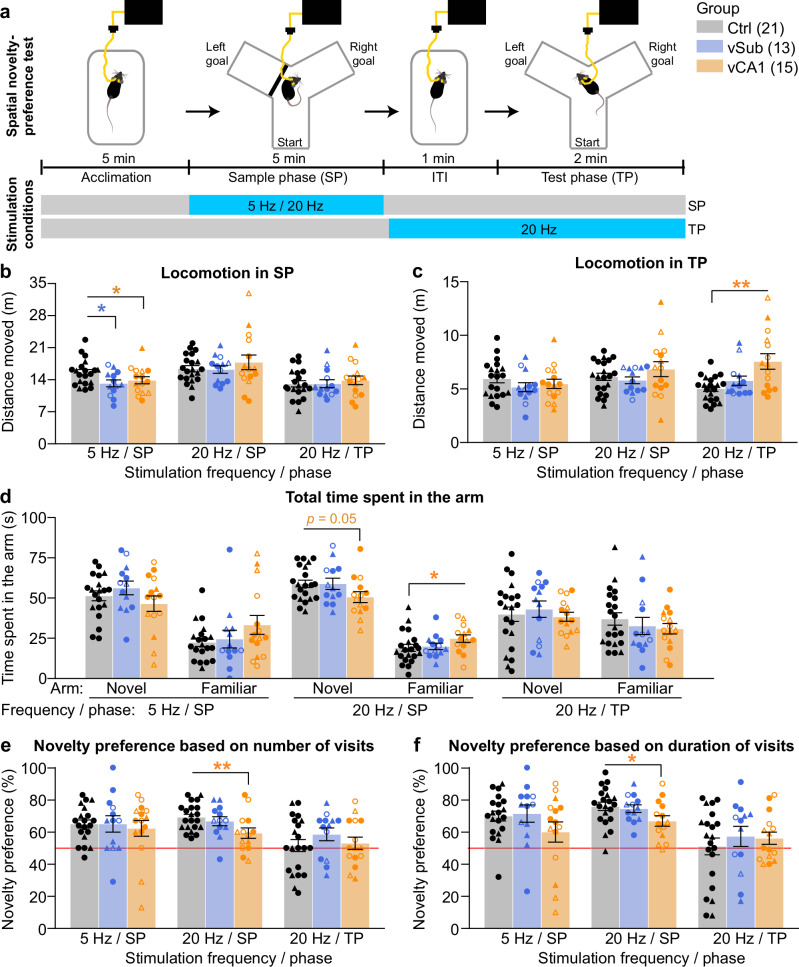
Impairment of spatial novelty-preference by optogenetic stimulation of vCA1. **a** Time-course of spatial novelty-preference tests conducted in the Y-maze with optogenetic stimulation either in the SP (5 Hz or 20 Hz) or in the ITI and TP combined (20 Hz only). **b**, **c** Locomotor activity expressed as distance moved in the whole SP (**b**) or TP (**c**). **d** Total time spent in each of the goal arms in the TP, separated by experiments with stimulation in the SP (5 Hz or 20 Hz) or TP. **e**, **f** Spatial novelty-preference index calculated from the number of entries into the two-goal arms (**e**) or the total time spent in such goal arms (**f**) as the ratio of (novel arm)/(both goal arms); red line represents chance level. Note that stimulation in the TP led to chance-level scores in all three groups, possibly due to optical distraction (failed experiment). Individual symbols represent individual subjects, whereby circles and triangles represent mice where the left or the right, respectively, hemisphere was stimulated; open symbols indicate mice with off-target expression in the vDG (not shown for Ctrl group), error bars represent s.e.m., asterisks indicate a significant difference in non-parametric paired comparisons to the control group (Ctrl, black; MWU-test). See Supplementary Tables 2–4 for parametric analyses. *N*-numbers are indicated in brackets for each group (**a**). One vCA1 mouse was excluded from the 20 Hz SP-stimulation experiment because it remained sitting in one arm. *p < 0.05; **p < 0.01.

Given the minor effects of vSub/vCA1 stimulation on exploratory drive and anxiety—compared to the strong effect of hyperlocomotion, especially with vCA1-activation (Fig. 1d, e)—we investigated the effect of vHC hyperactivity on another psychological process determining locomotor activity in an open-field: spatial short-term habituation, a form of spatial short-term memory assessable in the Y-maze, which we have previously found to be impaired with vHC-stimulation at 20 Hz^26^. In three separate experiments, conducted 2–3 weeks apart, we applied optical activation either in the 5 min sample phase (SP) (one experiment with 5 Hz, one with 20 Hz) in which animals memorize a spatial environment, or in the intra-trial-interval (ITI) and test-phase (TP) combined (1 + 2 min; 20 Hz only); in the TP a novel goal arm is accessible and usually preferred over the familiar goal arm (spatial novelty-preference) given intact short-term habituation (Fig. 4a). Locomotor activity itself was not further elevated by optical vSub stimulation, and was increased only by vCA1-stimulation in the TP (*p* = 0.005, MWU-test compared to Ctrl group; Fig. 4b, c). Unexpectedly, locomotion even decreased mildly with 5 Hz stimulation in the SP (Fig. 4b). Importantly, spatial novelty-preference (SNP) was impaired by inducing hyperactivity at 20 Hz in vCA1 (*p* < 0.05), but not in vSub (*p* > 0.5, MWU-test against Ctrl group; Fig. 4d, e) during the SP. As with locomotor activity (Fig. 1d, e), 5 Hz stimulation in the same phase had no effect (Fig. 4d, e). The experiment with stimulation in the TP failed, since all groups, including controls, exhibited a lack of novelty-preference, potentially due to optical distraction (Fig. 4d, e). (Note that a very low novelty-preference in controls during TP stimulation has also been observed in our previous study, whereas an impairment with vHC stimulation in the SP had been observed as well^26^).

### vCA1 hyperactivity impairs novel-object recognition (NOR)

**Fig. 5:**
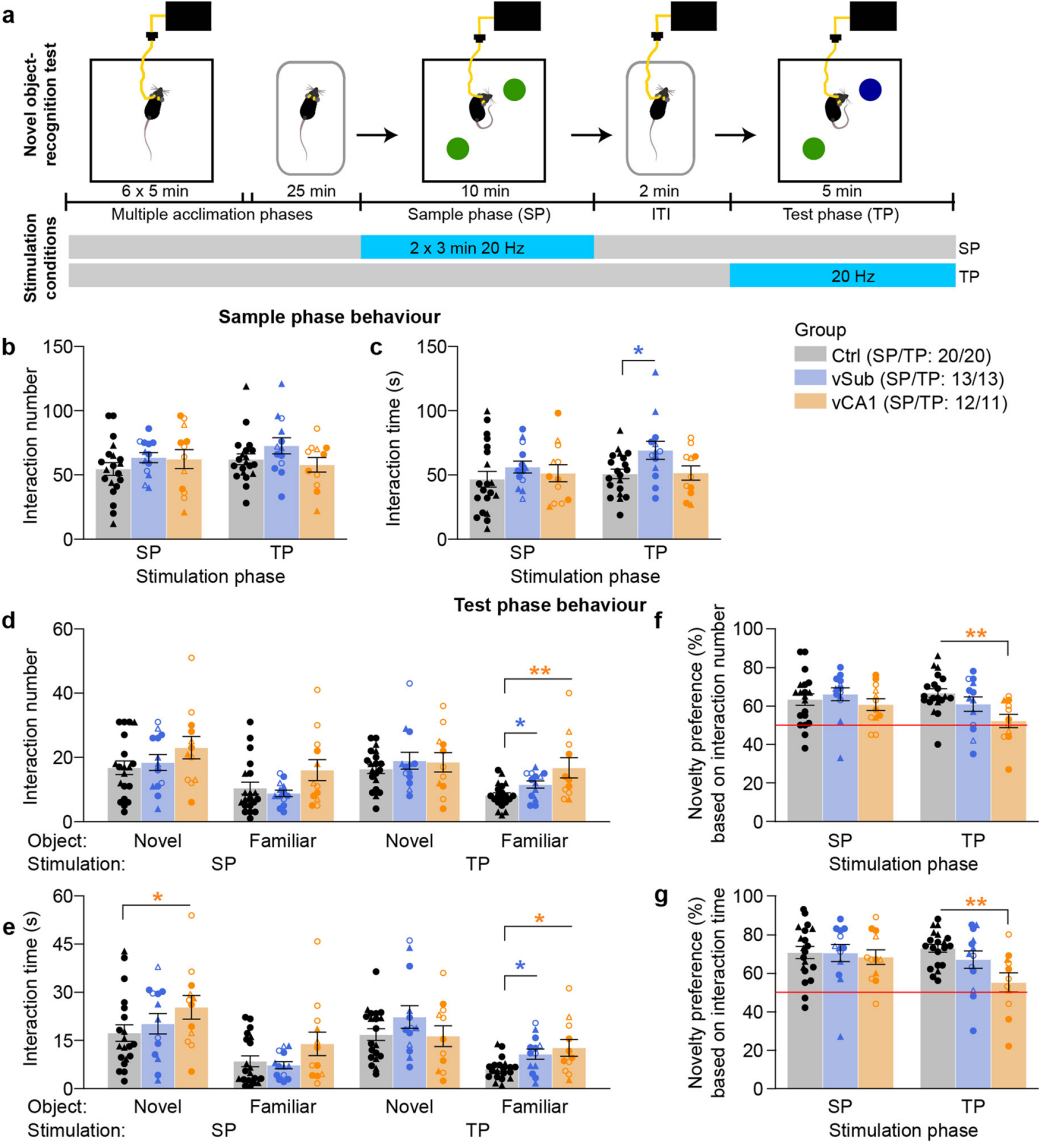
Impairment of object-related novelty-preference (novel-object recognition, NOR) by optogenetic stimulation of vCA1. **a** Time-course of NOR test conducted in an open field after multiple habituation phases. Twenty-hertz optogenetic stimulation (5 s-on/5 s-off) was delivered either in the SP or in the TP. **b**, **c** Number (**b**) and total time (**c**) of interactions with the objects in the SP, shown for experiments with stimulation in the SP or TP. **d**, **e** Number (**d**) and total time (**e**) of interactions with the objects in the TP, shown for experiments with stimulation in the SP or TP. **f**, **g** Novelty-preference index calculated from the number of object-interactions (**f**) or the total object-interaction time (**g**) as the ratio of (novel object)/(both objects); the red line represents chance level. Individual symbols represent individual subjects, whereby circles and triangles represent mice where the left or the right, respectively, hemisphere was stimulated; open symbols indicate mice with off-target expression in the vDG (not shown for Ctrl group); error bars represent s.e.m., asterisks indicate a significant difference in non-parametric paired comparisons to the control group (Ctrl, black; MWU-test). See Supplementary Tables 2–4 for parametric analysis. *N*-numbers are indicated in brackets for each group (legend in (**c**)) separately for experiments with stimulation in the SP or TP. Three vCA1 mice did not participate in the experiment, one further vCA1 mouse was excluded from the TP-stimulation experiment where it showed seizure-like behaviour and very low object interaction; one Ctrl mouse was excluded because it consistently showed almost no object interaction in both experiments (<3 s for both objects combined in TP). *p < 0.05; **p < 0.01.

Given the deficit in spatial short-term habituation, we wondered if the equivalent impairment would be inducible with object stimuli. We, therefore, conducted two instances of a novel-object recognition test, one with optogenetic stimulation in the SP, and another one with stimulation in the TP (both at 20 Hz; Fig. 5a). Stimulation during the SP, had no effect on the number or time of object interaction in either phase when directed to vSub, and it only caused a minor increase of object-interaction time in the test-phase when directed to vCA1 (*p* = 0.031, MWU-test; Fig. 5b–e). Importantly, it did not impair novel-object preference (*p* > 0.5; Fig. 5f, g). Optogenetically induced hyperactivity in vCA1 during the TP, in contrast, caused an increased interaction with the familiar object (*p* < 0.01; Fig. 5d, e), thereby impairing novel-object preference (*p* < 0.01, MWU-test against controls; Fig. 5f, g). While vSub-stimulation also caused a mild increase in the interaction with the familiar object (*p* < 0.05; Fig. 5d, e), NOR was not impaired (*p* > 0.5; Fig. 5f, g).

### vCA1 hyperactivity impairs working memory under certain conditions

**Fig. 6:**
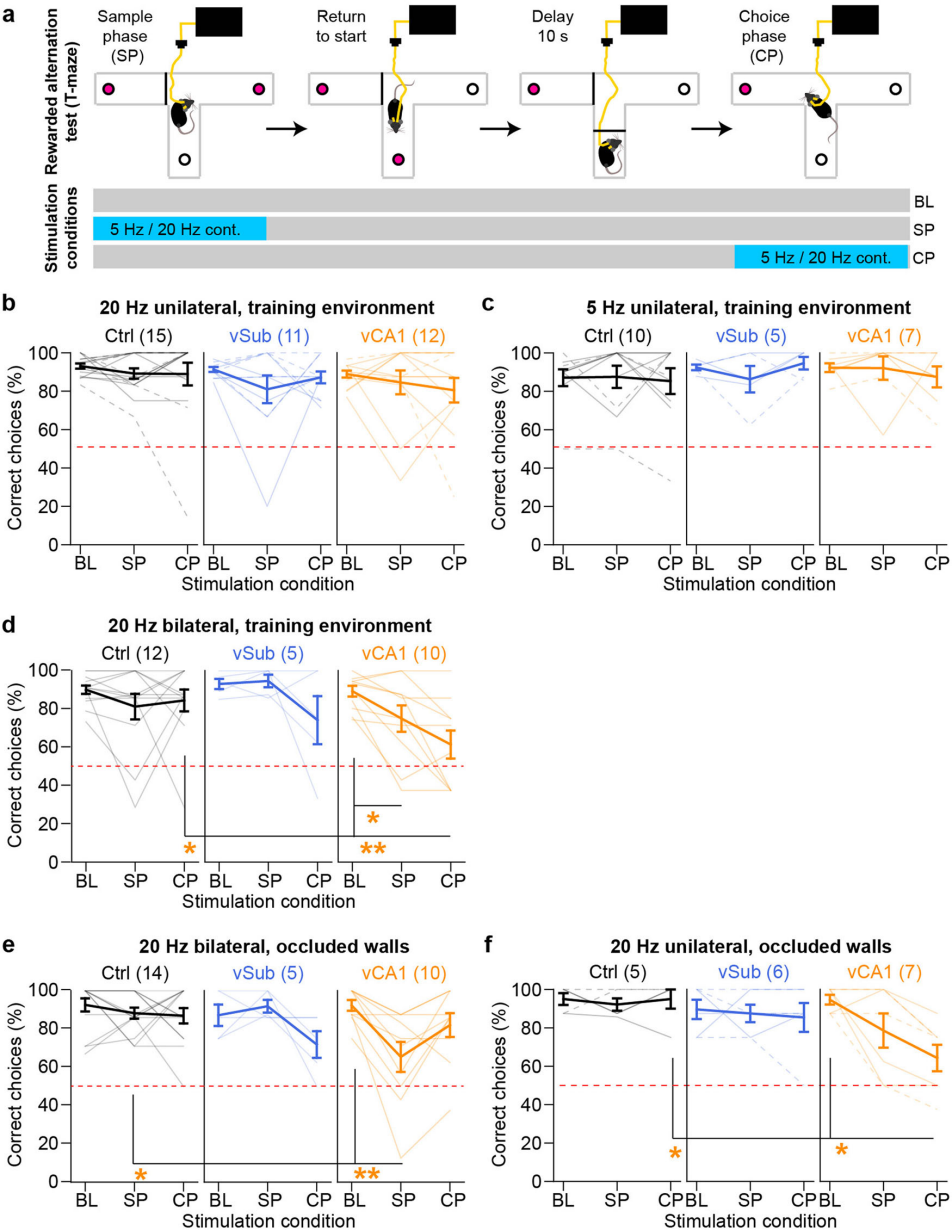
Impairment of spatial working memory by optogenetic stimulation of vCA1. **a** Time-course of a single trial of the T-maze rewarded alternation test, whereby optogenetic stimulation was either omitted (BL) or delivered continuously at 5 Hz or 20 Hz during the SP or the CP. **b**–**f** Working memory accuracy, expressed as %correct choices for distinct stimulation conditions: unilateral stimulation with 20 Hz (**b**) or 5 Hz (**c**), or bilateral stimulation with 20 Hz (**d**) in the familiar environment in which the animals have received their training sessions; 20 Hz bilateral (**e**) or unilateral (**f**) stimulation with occluded side-walls. *N*-numbers (stated in brackets above each sub-panel) in (**c**, **f**) are reduced because only batch 1 (**c**) or batch 2 (**f**) of the cohort conducted these experiments; *N*-numbers in (**d**, **e**) are reduced because only animals with bilateral expression were included in the analysis of bilateral stimulation experiments. Further reductions of *N*-numbers, compared to the original cohort, are due to insufficient participation in the task or lack of consistent reward consumption. Individual lines represent individual subjects, whereby solid and dashed lines represent mice where the left or the right, respectively, hemisphere was stimulated; error bars represent s.e.m.; red dashed line indicates chance level; asterisks within each sub-panel indicate a significant difference in pairwise within-subject comparisons of either one of the stimulation conditions compared to the BL condition (Wilcoxon signed-ranks test); asterisks in the Ctrl subpanels indicate non-parametric paired between-subject comparisons to the control group (Ctrl, black; MWU-test). See Supplementary Tables 2 and 4 for parametric analysis and Supplementary Fig. 3 for a display of the same data with an indication of mice with vDG off-target expression. *p < 0.05; **p < 0.01.

Given the detrimental effects of vCA1 hyperactivity on spatial and object-related short-term memory, we wondered if working memory was also affected. We trained mice in the T-maze rewarded alternation test of spatial working memory. In each trial, mice enter one pre-determined goal arm to earn a reward, then return to the start arm to earn another reward and spend a 10 s delay; subsequently, they enter one of the two goal arms whereby only the entry into the alternate goal arm is rewarded and counted as a correct choice (Fig. 6a). Three optogenetic stimulation conditions were tested on distinct test-days, separated by a training day: no stimulation throughout the trial (BL), stimulation during the SP, or stimulation during the choice phase (CP; Fig. 6a). To assess a potential impairment, we performed within-subject Wilcoxon signed-ranks tests comparing each stimulation condition (SP/CP) to performance under BL, within every group (in addition to standard MWU-tests comparing each experimental group to the control group). As in the other behavioural assays, we first probed the effect of a 20 Hz stimulation delivered unilaterally, but found no effect of stimulation in any phase or group (*p* > 0.1; Fig. 6b). In a subset of mice, 5 Hz unilateral stimulation was probed as well, but—expectedly—yielded no effect either (*p* > 0.2; Fig. 6c). Assuming that the natural activity in the non-stimulated hemisphere was somehow sufficient to enable spatial working memory, we repeated the experiment but with bilateral stimulation (animals that did not display sufficient or region-specific bilateral expression were excluded from analysis post-hoc). Indeed, 20 Hz bilateral stimulation of vCA1 in either SP or CP reduced performance compared to BL (*p* < 0.05, Wilcoxon-test; Fig. 6d). We also assessed the effect of the same manipulation, albeit with altered spatial cues using an occlusion of the previously transparent side walls of the maze. Again, we found a reduction of performance with vCA1-stimulation in the SP (*p* = 0.007), but not in the CP (Fig. 6e). Finally, in a subgroup of mice, we asked whether the change of spatial cues was sufficient to obtain an impairment even with just unilateral vCA1 stimulation at 20 Hz; in this case, performance was indeed significantly impaired with CP stimulation (*p* = 0.016), whereas a numerical performance drop with SP-stimulation did not yield significance (*p* = 0.141; Fig. 6f). It needs to be noted that negative results in both the latter experiment, as well as in most experiments in the vSub group need to be interpreted with caution given the low *N*-numbers resulting either from the exclusion of mice due to histological, behavioural or logistic reasons (e.g. lack of sufficient and specific bilateral expression, especially in vSub; insufficient participation of mice in experiments, testing of only a subgroup in experiments depicted in Fig. 6c, f). In fact, the vSub group repeatedly showed a numerical decrease in performance with bilateral stimulation in the CP (*p* < 0.2, Wilcoxon-test; Fig. 6d, e) which reached a trend-level effect in combination with occlusion of side-walls (*p* = 0.066). Overall, the experiments demonstrated that vCA1-hyperactivity either during memory encoding (SP) or retrieval (CP) can be detrimental to working memory performance, if it occurs bilaterally or if a reduced or altered set of spatial cues needs to be used to guide choices.

### vHC stimulation does not affect social interaction

**Fig. 7:**
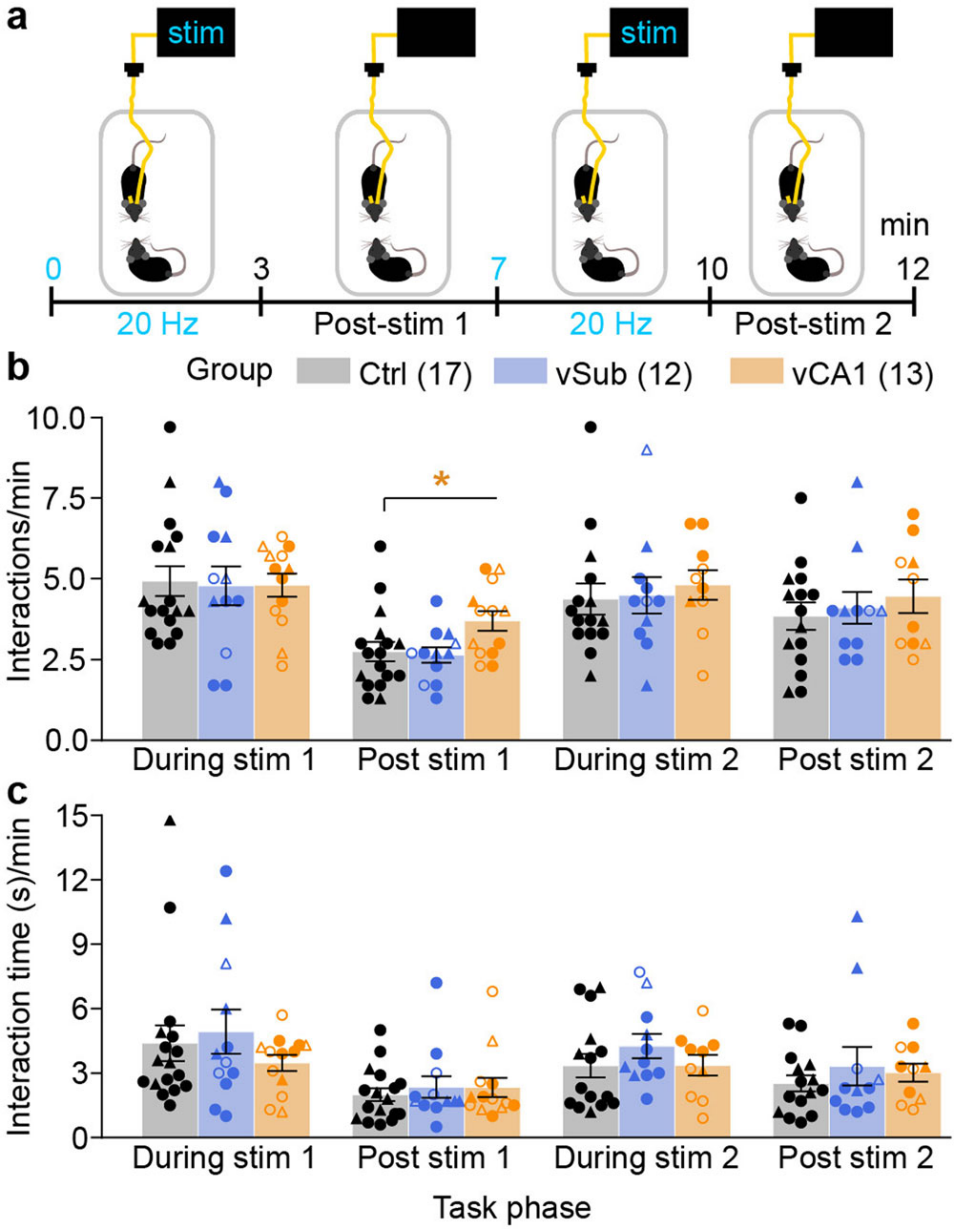
Optogenetic stimulation of vSub or vCA1 does not alter social interaction. **a** Time-course of the reciprocal social interaction experiment in a familiar open field with concomitant 20 Hz stimulation (5 s-on/5 s-off) in 1–3 min (stim1) and 8–10 min (stim2). **b**, **c** Number of interactions (**b**) and total interaction time (**c**) per minute in four phases of the experiment as indicated on the *x*-axis; Post-stim 1 and 2 refer to 4–5 min and 11–12 min, respectively. Individual symbols represent individual subjects, whereby circles and triangles represent mice where the left or the right, respectively, hemisphere was stimulated; open symbols indicate mice with off-target expression in the vDG (not shown for Ctrl group); error bars represent s.e.m., an asterisk indicates a significant difference in non-parametric paired comparisons to the control group (Ctrl, black; MWU-test). See Supplementary Table 2 for parametric analysis. *N*-numbers are indicated in brackets for each group (**b**); three Ctrl mice, two vCA1 mice and one vSub mouse did not participate in the experiment; one Ctrl mouse was excluded as an outlier. *p < 0.05.

Finally, we assessed the effect of unilateral vHC stimulation on a deficit of the negative symptom domain, sociability. Mice were introduced into a novel open field with an adult stimulus mouse of the same sex for 12 min, and optogenetic stimulation of 20 Hz was delivered in two 3 min-intervals (0–3 min and 7–10 min; Fig. 7a). No changes to the number or time of interaction were observed during either vSub or vCA1 stimulation (*p* > 0.1, MWU-test comparing to Ctrl-group; Fig. 7b, c). Interactions increased mildly after the first vCA1-stimulation (Fig. 7b), which may reflect lasting changes in the perceived salience of the environmental cues (as observed with the locomotor assay before, Fig. 1d).

## Discussion

The CA1 region of the human anterior hippocampus selectively displays abnormally elevated activity in prodromal high-risk individuals that later transition to psychosis^4^, but its causal role for symptoms that these patients display in the prodrome^20,43,44^ has so far remained elusive. We here demonstrated, that optogenetically induced elevated excitatory activity in the corresponding ventral CA1 region in mice can induce a range of deficits related to the control of selective attention and salience attribution, as well as to short-term and working memory, each of which is central to positive or cognitive symptoms, respectively, displayed in the prodrome and in overt schizophrenia^1,15^. In contrast, sociability, which is also impaired in prodromal individuals^44^, was not affected by vCA1-hyperactivity, although other tests of sociability are warranted in this model to confirm this conclusion, in future. Overall, our study confirms that elevated CA1-activity in the prodrome may be a direct cause of cognitive deficits in this phase^43^—in line with results with more broad vHC activation and disinhibition^1,24,27^. Moreover, deficits induced in assays of impaired short-term habituation (spatial and object-related novelty-preference, novelty-induced locomotor activity) make plausible a model in which elevated CA1-activity causes aberrant attribution of salience (due to impaired short-term habituation) which is suspected to drive the development of a delusional structure of thought and psychosis^1,45,46^. Since we only tested consequences of *acute* activation—not chronic hyperactivity, as observed in patients—further evaluation of experimentally induced chronic over-activation is warranted. Nevertheless, our paradigm does emulate increased hippocampal activity during the presentation of familiar stimuli as observed in patients^8^.

The impairing effects of vCA1-stimulation stand in surprising contrast to the limited effects of a selective increase of vSub activity—largely sparing the assessed cognitive deficits—given that vSub is considered to be the main projection target of vCA1 and the output station of the vHC. According to a prominent neuro-developmental account of schizophrenia^47^, this disease arises due to an iterative breakdown of inhibition in anterior CA1, which ensues hyperactivity in the anterior subiculum, which, in turn, is the main culprit for psychosis and potentially other symptoms of schizophrenia via dysregulation of the nucleus accumbens and ventral tegmental area (VTA) dopamine neurons^1^. The observation that anterior CA1 is hyperactive in prodromal individuals that later transition to psychosis, whereas anterior subiculum is hyperactive during or after the transition has occurred^3,4^, fits this model. The observed elevation of exploration in the locomotor activity and hole-board assays are in agreement with a resulting increase of dopaminergic signalling after vSub stimulation. However, the overall picture of our results suggests that a hyperactive vCA1 is the main cause of deficits in short-term memory and salience attribution. Consequently, these deficits are probably caused through an alternative output pathway, other than vSub, such as projections to the prelimbic cortex^48–50^ which have been demonstrated to be relevant for spatial working memory^49,51^, attention^52^, and also cognitive flexibility^53^. At the physiological level, CA1-activity in animals^54^ and hippocampal activity in healthy humans^8^ decreases with repeated exposure to initially novel, and therefore salient, stimuli, thereby mimicking—and potentially underlying^1,15^—the psychological process of short-term habituation by which perceived salience of increasingly familiar stimuli is gradually reduced, as detailed elsewhere^15^. In schizophrenia patients, this habituation of hippocampal activity is impaired^8^, as is the appropriate adjustment of perceived salience^45,46,55,56^. Modelling this hippocampal hyperactivity by acute optogenetic elevation in assays of short-term habituation in the present study has revealed that its mechanistic effect depends on the type of stimulus: SNP was impaired by stimulation in the sample-phase, whereas NOR was only impaired by stimulation in the test-phase (not in the sample-phase). Impaired novelty-preference due to stimulation in the sample phase implies that the habituation process itself—i.e. implicit memory formation (encoding)—is impaired by hippocampal hyperactivity, making the familiar space to be perceived as salient as an actual novel space in the later test phase. In line with this interpretation, in the test phase, exploration of the familiar arm was elevated and that of the novel arm was reduced (Fig. 4d), indicating an increased perceived salience of the familiar relative to the novel arm. In contrast, for object stimuli, an impairment of novelty-preference was only caused by stimulation in the test phase, which implies a direct disturbance of accurate familiarity-based salience assignment by vCA1 overactivation, i.e. a failure of implicit memory retrieval. Importantly, novelty-preference was mainly impaired by increased interaction with the familiar object—as opposed to decreased interaction with the novel object—supporting the prediction that hippocampal hyperactivity leads to a failure specifically of a habituation-based salience assignment. In addition, stimulation in the sample phase of the NOR test also increased interaction with the novel object and, numerically, with the familiar object (*p* < 0.2) in the test phase, without affecting novelty-preference; this may indicate an additional failure of appropriate salience assignment by over-generalization whereby objects appear as generally more salient after the previous hippocampal stimulation during object-exploration. This, again, contrasts with SNP, where visits to the novel arm in the test phase were actually decreased by stimulation in the sample phase. However, data from both tests support the notion that increased hippocampal activity during perception of familiar stimuli—as observed in patients with schizophrenia^8^—elevates the perceived salience of such stimuli beyond what would be appropriate given their relative familiarity and beyond what it would be under conditions of intact stimulus-specific short-term habituation. Although the effect of optogenetic stimulation in the test phase of the Y-maze test on SNP could not be evaluated as also control mice showed no SNP in this scenario, the comparison of hyperlocomotion effects across assays—which are observed after the mice could familiarize themselves with the environment for a few minutes (open-field and test-phase of the Y-maze) but not in a novel space (EPM and sample-phase of the Y-maze)—further supports the notion that hippocampal hyperactivity specifically disrupts habituation-mediated reduction of salience attribution to familiar spatial stimuli.

As a caveat of our results, it needs to be noted that our univariate, pairwise non-parametric analysis, chosen because of the data distribution, does not allow us to draw strong conclusions regarding differential effects of either manipulated region (vCA1 vs vSub) and may lead to a higher probability of false-positive results. Given that ANOVA is relatively robust against violations of assumptions of normality, we also provided parametric analyses of all important behavioural variables (Supplementary Tables 2–4), which largely support the conclusions inferred from non-parametric tests. A notable exception is that an increase in hole exploration by vSub-stimulation did not reach significance. Importantly, repeated-measures ANOVA confirmed the effect of vCA1-stimulation in the test phase of the NOR assay as selectively increasing the exploration of familiar objects to a level that is statistically indistinguishable from the exploration of novel objects (Supplementary Table 3). Also, even though the number of indicative trend-level results is small (Supplementary Table 2)—the possibility of false-negative results, especially in the vSub-group, cannot be fully excluded, given that *N*-numbers may be too low to ensure sufficient statistical power for such variable behavioural data with non-parametric or even parametric analysis. The most likely candidates of effects that may be more detectable with testing of larger groups in future experiments include potential decreases of anxiety by stimulation of either vSub or vCA1 (Fig. 3c–e), and detrimental effects of vSub-stimulation on novel-object preference (Fig. 5f, g) and rewarded alternation (bilateral stimulation in the CP; Fig. 6d, e). Also, false-negative results may have arisen due to incomplete transduction of the total volume of each sub-field, which was a necessary compromise to minimise off-target expression in other areas. A further potential confound may arise from the experimental history of the subjects: since all tests—including the vHC stimulation at different frequencies (5 Hz and 20 Hz)—were conducted sequentially in the same animals, prior stimulation of neurons or the experience of stimulation at different frequencies may have affected the responsiveness of vHC cells to later stimulations and the conscious perception of their effect, and thereby their behavioural consequences. Furthermore, we cannot fully exclude a contribution of the activation of the outer blade of the dentate gyrus to the observed behavioural deficits, which was often mildly co-transfected in vCA1 animals (see Fig. 1a; Supplementary Figs. 1 and 2; and Supplementary Table 1), although not targeted by the optical fibre. Whereas *N*-numbers are too low for statistical assessment of the sub-groups with vs without vDG off-target expression, a qualitative inspection of the plots where these are distinguished by different symbols (Figs. 1, 3–5, and 7; and Supplementary Fig. 3) suggests that the vDG-expression has no influence on the obtained results in assays of short-term habituation (Figs. 4–5), anxiety (Fig. 3), and social interaction (Fig. 7). In optically induced hyperlocomotion (Fig. 1) and two of the T-maze conditions (20 Hz bilateral stimulation in training environment and 20 Hz unilateral stimulation with occluded walls; Supplementary Fig. 3) however, animals with vDG co-expression appeared to be more strongly affected compared to vCA1-mice without such off-target expression. These potential differences would not be unexpected given the overall stronger excitatory drive delivered to vHC, and they remain to be examined in future studies. In contrast, qualitative observations across tasks (Figs. 1–7) suggest that the choice of stimulated hemisphere appears to have no influence on the overall results, despite the fact that BL hippocampal hyperactivity is lateralized to the left in human prodromal patients^4^.

In summary, our results indicate that abnormal hyperactivity in the ventral CA1 may cause deficits in short-term memory and salience processing that are central to schizophrenia and its prodrome and warrant the exploration of early intervention strategies that target this cellular abnormality.

## Methods

### Animals

In total, 72 adult male B6.Cg-Tg (Camk2a-cre)T29-1Stl/J (CamKIIα-*Cre*, originally obtained from The Jackson Laboratory, Maine, US; bred in-house)^57^ were used for this study. Sample sizes were estimated based on results from the Y-maze novelty-preference test in our previous study on optogenetic stimulation of the vHC^26^, resulting in a suggested *N* = 26 with a 30–50% exclusion rate when assuming analysis with a *t*-test for groups with unequal variance. Assuming a larger exclusion rate due to the angled injections for vSub-transductions (see below), we operated 29 vSub, 22 vCA1 and 21 Ctrl animals to arrive at 13–18 mice after histology-based post-hoc exclusions. Due to logistic constraints, given the size of the cohort, mice conducted the experiments described below in two separate batches, each containing subjects of every experimental group stated below. Animals were group-housed in Type II-Long individually ventilated cages (Greenline, Tecniplast, DE), enriched with sawdust, sizzle-nest^TM^, and cardboard houses (Datesand, UK), and maintained at a 13 h light/11 h dark cycle. Food and water were available ad libitum, except for a period of scheduled feeding for the T-maze rewarded alternation task, see below. CamKIIα-*Cre* mice were genotyped for *Cre* using primers 5’-GCGGTCTGGCAGTAAAAACTATC-3’ and 5’-GTGAAACAGCATTGCTGTCACTT-3’ (100 bp product), and for the wildtype locus using primers 5’-CTAGGCCACAGAATTGAAAGATCT-3’ (324 bp product). All experiments were performed in accordance with the German Animal Rights Law (Tierschutzgesetz) 2013 and were approved by the Federal Ethical Review Committee (Regierungspräsidium Tübingen) of Baden-Württemberg, Germany (licence number TV1324).

### Surgical procedure

Before any behavioural assessment, mice aged 4.0 ± 1.5 months (mean ± S.D.) underwent stereotaxic surgery for bilateral infusion of the AAV viral vector and bilateral implantation of the fibre-optic cannula. Animals were anaesthetized using isoflurane (AbbVie, G), and received s.c. injections of analgesics (0.08 mg/kg buprenorphine, Bayer, G; 5 mg/kg meloxicam, Boehringer Ingelheim, G), and local scalp anaesthesia (2 mg/kg bupivacaine, AstraZeneca, UK) before placement in a manual, digital, stereotaxic frame (World Precision Instruments, US) with non-rupture mouse ear bars. The body temperature was stabilized using a feedback-controlled heating blanket (Harvard Apparatus, US) and the anaesthesia was maintained with 1.2–1.5% isoflurane. The following stereotaxic coordinates (AP and ML from bregma, z from pia), angle (from vertical), and volumes were used for bilateral transductions of the stated areas (see Fig. 1a, b); vCA1: AP −3.1, ML 3.5, z 3.1 (150 nl) and 2.8 (80 nl), angle 0°. vSub: AP −3.6, ML 4.4, z 3.3 (80 nl), angle 22° inward. Bevelled metal needles (WPI, US) of 35 gauge diameter were used with the bevel facing lateral (vCA1) or posterior (vSub), respectively, in combination with a glass 10 μl precision syringe (WPI) and a motorized pump controller (Micro4, WPI). Upon insertion, needles were moved 40–50 μm beyond the first infusion site and then retracted to the actual coordinate to create a small pouch for the infused suspension. Viral suspensions were infused at 50 nl/min and needles were left in place for 6 min after the final infusion, before slowly moving the needle up by 100 μm to wait there for another 3 min, followed by a slow withdrawal; for vCA1, the needle was left in place for 3 min at the first drop side, before moving it up slowly to the second infusion site. These infusion parameters were optimized to render the transfection of the target region as selective as possible. Groups vCA1 and vSub received the vector AAV-EF1α-FLEX-Chronos-eGFP of serotype AAV8 at a titre of 5.1 × 10e12 vg/ml (University of North Carolina vector core, UNC, NC, US), whereas the Ctrl group received an AAV-hSyn-FLEX-eGFP vector at a titre of 3.4 × 10e12 vg/ml of either AAV5 (UNC) or AAV8 serotype (University of Zürich viral vector facility, UZH-VVF, CH). The final titre was obtained by dilution in 5% sorbitol/PBS (Sigma, G), wherever the original titre was higher. After viral transfection was completed on both hemispheres, fibre-optic cannulas with an optic fibre of 200 μm diameter (Thorlabs, DE), freshly cut to an optimal length with a diamond knife, were slowly inserted vertically into the following coordinates; vCA1: AP −3.1, ML 3.5, z 2.6; vSub: AP −3.6, ML 2.9, z 3.8. Fibre optic cannulae were fixed to the skull with light-curable dental adhesive (Breeze^TM^, Pentron, US), and the skin of the anterior and posterior end of the implant was sutured together to cover the rim of the implant. Mice received post-operative monitoring for 7 days, and an s.c. injection of the analgesic meloxicam (Metacam, 5 mg/kg, Boehringer Ingelheim, G) on the first 3 days.

### Optical stimulation

During behavioural testing, described below, mice received mostly unilateral optical stimulation; only during certain paradigms in the T-maze, simultaneous bilateral stimulation was used. A 470 nm diode laser (200 mW output, stability < 5% RMS/4h; LRD-0470-PFFD-00200-05; Laserglow, CA) connected to an optical fibre (Thorlabs, DE) via an FC/PC collimator (Laserglow) was used as a laser source and was directly gated through an OTPG-4 microcontroller (Doric lenses, CA), triggered by ANY-maze (ANY-maze) via an ANY-maze interface (AMi) system (Stoelting, US). For locomotor experiments, where two mice were tested at the same time, the optical fibre protruding from the collimator was connected to a channel splitter (Doric Lenses). In any case, optical fibres were connected to a low-friction single-channel or (for bilateral stimulation) channel-splitting rotary joint (Doric Lenses) that was attached above the centre of the behavioural apparatus. Thin-walled, flexible optical fibres with a simple ferrule-termination (Doric Lenses or Thorlabs) were connected to the lower end of the rotary joint and attached to the ferrule of the fibre-optic cannula implanted on the animal via a zirconia sleeve (Thorlabs). All optical fibres in the pathway had a diameter of 200 μm. Optical stimulation was done at either 5 Hz or 20 Hz with pulses of 5 ms duration. Whereas 5 Hz stimulation was delivered continuously, 20 Hz stimulation was delivered alternating 5 s with and 5 s without stimulation (5 s-on/5 s-off), except for the T-maze test, where it was also done continuously given the short duration of stimulation. Optical output power measured at the ferrule that was subsequently connected to the implanted fibre-optic cannula ranged between 2.5 mW and 5 mW and was adjusted for every hemisphere of every animal individually, as described below. Stimulation was delivered unilaterally in all experiments, and only bilaterally in the T-maze due to a lack of an initial effect with unilateral stimulation. Unilateral (as opposed to bilateral) stimulation was chosen for multiple reasons: firstly, hippocampal hyperactivity is lateralized in schizophrenia patients, as well, affecting mostly the left hemisphere^4^. Hence unilateral stimulation matches the clinical phenotype more accurately whereas bilateral stimulation could be too intense and disrupt neural processes much more than the clinically observed hyperactivity would. Secondly, the aim of the study to target vCA1 and vSub specifically implied that a substantial number of animals would have to be excluded based on post-mortem histological assessments due to off-target expression in other subfields. Demanding accurate viral expression in both hemispheres would vastly increase the exclusion of mice, which appears unethical given that unilateral stimulation appears to be sufficient to cause schizophrenia-related deficits, such as a hyperdopaminergic state^26,40^. Thirdly, in our previous study^26^, a small number of animals had to be excluded due to seizures, likely resulting from strong off-target expression in CA3 and/or the dentate gyrus. Therefore, restricting the manipulation to unilateral stimulation was expected to decrease the probability of stimulating a hemisphere with such off-target expression or of causing over-stimulation in general. Note that stimulation in the T-maze is much shorter (a few seconds compared to 3–5 min) and hence entails a lower risk of over-stimulation even with bilateral stimulation.

### Behavioural procedures

Behavioural testing was done blind to the subgroup identity of the mice. All testing was done in the light phase, starting at least 1 h after the onset of the light in the holding room and ending no later than 1 h before the onset of the dark phase. All behavioural tests and stimulation conditions were done in the same group of mice.

### Optogenetically induced hyperlocomotion

After the postoperative period, but before actual testing started, the optimal hemisphere and optical power were determined using the readout of stimulation-induced locomotor activity as described previously^26^. Briefly, mice underwent several test sessions of 24 min exploration in a novel clear plastic cage (425 × 266 × 185 mm; Eurostandard Typ III, Tecniplast, DE) filled with clean sawdust, with at least 2 days in between consecutive sessions. Ambient light levels were 100 lux. In each session, one hemisphere was stimulated individually at 20 Hz for 3 min from 16 min to 18 min (6th out of 8 3 min intervals). Output power was chosen at 2.0 mW in the first session, and it was increased in 0.5–1.0 mW steps until locomotion during stimulation was >115% of the average of the locomotion in the three preceding intervals 3–5 (7–15 min). Once this was observed, the same power was tested again in the subsequent test with this hemisphere, and it was determined as the final power, if an increase of >115% was observed again. Otherwise, the power was further increased as described before in subsequent sessions until two consecutive sessions with such increase were found for a given power value and hemisphere. Tested hemispheres alternated between test sessions, and the hemisphere where a reliable locomotion increase was observed first, was chosen as the primary target for unilateral stimulation. For bilateral stimulation during the T-maze (see below), the same power was used for both hemispheres. Control animals were taken through the same procedure, but the final optical power was chosen to approximately match the distribution of power values in the vSub and vCA1 groups. Movement was tracked using beam-break counts (PanLab, FR).

For a final analysis of locomotion increase, animals were tested in a novel environment and another 24-min session was conducted in cages with the same dimensions and saw-dust filling using the final stimulation power and 20 Hz stimulation-frequency, unilaterally. The experiment was repeated with a 5 Hz stimulation frequency, no earlier than one week after the first experiment, using a clean box placed at a different position in the rack and filled with fresh sawdust, so that it constitutes a novel environment just as for testing with 20 Hz. CCTV cameras (Sentient, GB) installed centrally above the open-field cage were used to monitor the animals. Videos from two cage stations were assembled into a single image frame through a CCTV system (Dahua Inc, China), digitized through an A/D converter (TheImagingSource, DE), and analysed by computer-based tracking with ANY-maze (Stoelting, IE). Data was analysed in 3-min intervals in terms of *absolute* distance moved per interval and of *relative* locomotion whereby distance moved during intervals 6 (stimulation) and 7 (post-stimulation) where normalized to the average distance moved in intervals 3–5. After finalization of the behavioural test battery described in this manuscript, further hyperlocomotion experiments were conducted with pharmacological modulation, but failed due to high variability of locomotion across mice (results not shown).

### Hole-board exploration

Testing was conducted in a hole board where poking into holes was detected with infra-red break-beams (PanLab) and recorded in the ActiTrack software (PanLab) at light levels of 100 lux. The area was restricted to 35 × 35 cm by a transparent Perspex wall of 20 cm height; the area contained 16 poke-holes of 12.5 cm centre-to-centre distance, 2.5 cm diameter, and 2.5 cm depth. Mice were tethered unilaterally to an optical fibre and explored the hole-board area for 15 min. The exploration period was analysed in five 3-min intervals and optical stimulation was conducted at 20 Hz in the fourth interval (10–12 min). Due to large animal-to-animal variation, the number of poke-holes in every interval was divided by the number of pokes performed in the middle interval 3 (6–9 min), which was also the interval immediately preceding the stimulation (BL).

### Elevated plus maze (EPM)

In order to assess exploratory drive in relation to unconditioned anxiety, an EPM made from grey plastic was used at ambient light levels of 80 lux in the centre of the maze, as previously described ref. ^26^. The +-shaped maze had two opposite closed arms and two open arms (35 cm long and 7 cm wide), elevated 75 cm above the ground. After 5 min of habituation in a novel plastic cage (Type II-Long cage, Greenline, Tecniplast) with sawdust in the test room, mice were tethered to the optical fibre and placed in the centre of the maze facing an open arm, whereby the optical stimulation (20 Hz, unilateral) was started ~10 s before. Exploration and stimulation occurred for 5 min. The position of the animal was tracked using a CCTV camera (Sentient) and ANY-maze (Stoelting), and zone transition was determined according to the position of the centre of the animal’s body. The preference for the open arms was calculated as the ratio of the total time in open arms and the time spent on all arms combined (disregarding the time spent in the centre).

### SNP in the Y-maze

SNP served as an indicator of spatial short-term memory mediated by spatial short-term habituation and was conducted as previously described^26^ at ambient light levels of 80 lux. Briefly, mice were tethered unilaterally and introduced to a novel plastic cage (Type II-Long, Greenline, Tecniplast) with saw-dust, for habituation for 5 min. Subsequently, mice were transferred to the end of the start arm of a Y-shaped maze made from transparent Perspex® (l × w × h dimensions of each arm in cm: 30 × 8 × 20) and filled with approx. two to three centimeter of saw-dust mixed with dirty bedding from an unfamiliar mouse cage. Mice were left to explore the start arm and one of the goal arms for a 5 min SP. The other goal arm (identity counter-balanced within each sub-group but maintained to be the same across the repetitions of the experiment for each animal) was shut off by a guillotine door made from grey, non-transparent PVC. Subsequently, mice were removed back into the plastic cage for an ITI of 1 min during which the guillotine door was removed and the saw-dust was mixed across all three arms. Animals were then reintroduced to the maze and were left to explore all three arms for a 2-min TP. The TP was chosen to be shorter than the SP to avoid confounding of the SNP score by habituation to the novel arm during the (longer) TP itself. The position of the animal was tracked using a CCTV camera (Sentient) and ANY-maze (Stoelting) and zone transition was determined according to the position of the centre of the animal’s body. The preference for the novel arm (accessible only during the TP) was calculated based on both the number and the total duration of visits using the SNP ratio of visits to the novel arm/visits to both goal arms combined. The SNP-preference test in the Y-maze was conducted once with 20 Hz stimulation in the SP (5 min), once with 20 Hz stimulation in the combined intra-trial-interval and TP (3 min), and once with 5 Hz stimulation in the SP (5 min), in that order. The length of the stimulation was determined by the length of the SP, ITI, and TP phases, and the ITI and TP were combined for stimulation to provide a possibly stronger disruptive effect (affecting maintenance and retrieval of the memory; see ref. ^26^) and match the stimulation duration in the SP more closely. In batch 1 of the cohort, a place-preference test was conducted in the Y-maze, in addition, after the last SNP-testing, analogous to our prior study, ref. ^26^ (results not shown). Furthermore, after the finalization of the behavioural test battery described in this manuscript, a further Y-maze experiment was conducted with pharmacological modulation but failed due to insufficient spatial exploration in many mice (results not shown).

### NOR

Preference for a novel object served as an indicator of short-term memory for objects mediated by object-related short-term habituation^15^. Animals were first accustomed to the square open field (dark-red floor, grey PVC walls of 25 cm height, 40 cm length and width) in six 5-min sessions distributed over the two days before testing. On the day of testing, mice were habituated to a holding cage for ca. twenty-five minutes, then tethered to the optical fibre and introduced to the familiar open field in which two copies of the same unfamiliar object were placed (SP). After 10 min, the mouse was removed from the open field into the holding cage for a 2-min ITI. At that time, one object was replaced by an identical, clean copy of the SP objects and the other one by a novel object. Objects were beverage containers differing in texture, shape, material and colour (green Powerhouse® can and blue Powerade® plastic bottle at the first run; black Coca Cola® glass bottle and yellow apple juice plastic bottle at the second run) and their choice as novel vs familiar object, as well as the position of the novel object were counterbalanced within each group. After the ITI, a 5-min TP followed during which mice explored the two new objects in the open field. Light levels were 100 lux at the centre of the open field. Mice conducted the NOR test twice, approximately four months apart; in one instance 20 Hz optogenetic stimulation was applied during the SP, namely at 1–3 min and 5–8 min (3 min each), whereas in the other instance stimulation was conducted throughout the 5 min TP; the stimulation phase was counter-balanced within every group. Their movement was video-monitored and their interaction with objects was scored manually from videos. Novelty-preference was analysed by calculating the time spent interacting with the novel object divided by the sum of the time spent with either object. Additionally, the same preference score was calculated based on the number of interactions.

### Rewarded alternation test of spatial working memory in the T-maze

The rewarded alternation task in the T-maze was used as a delayed non-matching to position (DNMTP) paradigm of spatial working memory and was conducted as previously described in refs. ^58,59^. The T-shaped maze (W 10 cm, L 40 cm, and H 10 cm) featured a red PVC floor and transparent Perspex walls with metal food wells at the end of each goal arm. Light levels were 100 lux at the T-junction. A 2:1 dilution of condensed milk (‘Ja’, REWE, G) in drinking water was used as a reward. Before the first testing day, mice were food-restricted to attain and maintain 85–90% of their free-feeding BL weight, and they were habituated to the maze and the consumption of the reward, first in groups (with cagemates, 3–5 min) and then individually (3 min). Such short habituation sessions were repeated over the course of 2–4 d until the mice consumed the reward reliably. The day after the last habituation session, training of the T-maze alternation paradigm was conducted with 5–10 massed trials per day for 7 days (batch 2 of the cohort) or 11 d (batch 1) and with the mouse being tethered to the optical fibre. Training days differed between the cohorts, because several mice in cohort 1 ran slower, so that ten trials/day could mostly not be achieved; the days were varied to roughly match the number of received training trials. Mice that did not run in the maze or consume the reward reliably were excluded from further training and testing in the T-maze experiment. An individual trial consisted of a sequence of SP, 5 s delay, the CP, and a 20 s ITI before the subsequent trial. In the SP, subjects were placed in the start arm and were allowed to enter a pseudo-randomly determined goal arm to consume the reward, while the alternate arm was blocked. After gaining the reward in the SP, the mouse was briefly removed from the maze for the delay phase during which the barrier blocking the previously inaccessible (novel) goal arm was removed. In the subsequent CP, the mouse was reinserted into the start arm and was left to choose between the previously unvisited rewarded and the previously visited unrewarded arm. Working memory performance (accuracy) was determined as the ratio of correct choices and the number of trials per session. For optogenetic modulation, different stimulation paradigms were conducted, each with a 10 s delay and a 20 s ITI, in the following order; in batch 1 of the cohort: 20 Hz unilateral, 5 Hz unilateral, 5 Hz bilateral, 20 Hz bilateral, 20 Hz bilateral in a different environment, 20 Hz bilateral with occluded side walls; in batch 2 of the cohort: 20 Hz unilateral, 20 Hz bilateral, 20 Hz bilateral with occluded side walls, 20 Hz unilateral with occluded side walls, and 5 Hz bilateral with occluded side walls. The sequences differed, because the overall number of testing sessions was logistically limited and the 5 Hz protocols without occluded side walls did not reveal any impairment in batch 1, and the testing in a different environment in batch 1, was superfluous given the protocol with occluded side walls. Due to these omissions in batch 2, the two last testing protocols (20 Hz unilateral and 5 Hz bilateral with occluded side walls) could be added to their sequence, which had not been conducted in batch 1. In every case, stimulation was conducted in either the SP or the CP, on separate days, and the order of the stimulation days was counterbalanced within every group. Test days were always interleaved with training days during which the side walls remained transparent. For testing paradigms with transparent side walls, performance on such intermittent training days was used as a non-stimulation BL condition for within-subject statistical comparison to sessions with optogenetic modulation. For testing paradigms with occluded side walls, the non-stimulation condition was treated as a third experimental condition in the counter-balancing scheme on the test days themselves; i.e. these paradigms involved three test days.

### Reciprocal social interaction

In the reciprocal social interaction test, the test mouse was exposed to a novel stimulus mouse which was an adult, but younger mouse of the same strain and sex, in a familiar open-field (dark but transparent Type III cage; Tecniplast, G) filled with familiar sawdust, for 12 min. Mice familiarised themselves with the open field during a habituation of 30 min on the preceding day, and the specific cage and sawdust of each mouse were retained for the testing day. Unilateral optogenetic stimulation was applied during two episodes of 3 min each, namely in 1–3 min and 8–10 min. Light levels were 100 lux.

### Histology

After the behavioural test battery, animals were given an overdose of ketamine/medetomidine (≥200 mg/kg ketamine, Zoetis, G; ≥2 mg/kg medetomidine, Pfizer, US) and perfused with 0.01 M phosphate-buffered saline (PBS) followed by 4% PFA/PBS. The brains were rapidly removed and then stored in 4% PFA/PBS overnight before placement in 20% sucrose for dehydration before sections were cut at 60 μm thickness on a vibratome (VT1000, Leica, Deerfield, IL, USA). Every second section was stained with DAPI (10^−4%^ w/v) for 30 min, washed with PBS twice and mounted on glass slides. A Leica DM6B epifluorescence microscope (Leica, G) was used to scan the slides with a 5× objective and determine virus expression offline. Animals were only included in the dataset if they showed expression in the target structure (vSub or vCA1) in the hemisphere that was primarily stimulated, but not in the brain region of the other group (vCA1 or vSub). About half of the vCA1 mice and three of the vSub mice also showed expression in the dendritic layer of the lateral portion of the dentate gyrus (Supplementary Fig. 1 and 2 and Supplementary Table 1) and are indicated by open symbols in all bar plots throughout the manuscript. Minor off-target expression in adjacent regions was observed in a small number of animals, as indicated in Supplementary Table 1. eGFP-transduced control animals were not excluded based on expression patterns.

### Statistics and reproducibility

Behavioural data was analysed using SPSS 26.0 or 29.0 (IBM, NY, US). Data from each of the two experimental groups (vCA1, vSub) was compared to the control group with a two-sided MWU-test (see the summary of results in Supplementary Table 2). Non-parametric testing was used due to the non-normal distribution of many datasets. We nevertheless provide results of the corresponding parametric analysis with one-way ANOVA and Dunnett’s post-hoc test as Supplementary Table 2 and, where applicable, with repeated-measures ANOVA with Sidak post-hoc tests as Supplementary Tables 3 and 4 for information. All bar and line graphs display mean ± s.e.m. and data from individual mice, as indicated.

## Supplementary information

Supplementary Information

## Data Availability

All raw data for behavioural experiments can be obtained from the corresponding author upon reasonable request.
