# Supplementary material for: Acute optogenetic induction of the prodromal endophenotype of CA1 hyperactivity causes schizophrenia-related deficits in cognition and salience attribution
Source: Schizophrenia (Heidelb). 2024 Oct 8;10(1):90. doi: 10.1038/s41537-024-00513-w (PMC11461789; doi:10.1038/s41537-024-00513-w)
Supplement: Supplementary file 1 — Supplementary Information [file 41537_2024_513_MOESM1_ESM.pdf]

Supplementary Information

Acute optogenetic induction of the prodromal endophenotype of CA1 hyperactivity causes schizophrenia-related deficits in cognition and salience attribution

Sampath K.T. Kapanaiah[1], Christina Grimm[1,2], Dennis Kätzel [1,*]

[1] Institute of Applied Physiology, Ulm University, Ulm, Germany

[2] Present address: School of Engineering, Neuro-X institute, EPFL, Lausanne, Switzerland; CIBM Center for Biomedical Imaging, Lausanne, Switzerland

Running title: Cognitive processing impaired by hippocampal hyperactivity

* Correspondence: dennis.kaetzel@uni-ulm.de; +49 731 500 33770; Fax +49 731 500 33779; Institute of Applied Physiology, Ulm University, Albert-Einstein-Allee 11, 89081 Ulm, Germany

Supplementary Tables

		unilaterally stimulated hemisphere					Other hemisphere (if used for bilateral stimulation)				
Group	ID	hemisphere	target expression	off-target expression	AP-anterior	AP-posterior	used	target expression	off-target expression	AP-anterior	AP-posterior
vSub	vSub_01	**L**	vSub	vDG	3.8	3.9	no	-	-	-	-
	vSub_02	**L**	vSub	(weak ant. PMCo/PLCo)	3.4	3.9	no	-	-	-	-
	vSub_03	**L**	vSub	none	3.3	3.7	no	-	-	-	-
	vSub_04	**L**	vSub	none	3.6	3.9	no	-	-	-	-
	vSub_05	**L**	vSub	vDG, vCA3	3.3	3.9	**yes**	vSub	vDG, vCA3	3.3	3.9
	vSub_06	**L**	vSub	none	3.8	3.9	**yes**	vSub	none	3.8	3.9
	vSub_07	**L**	vSub	none	3.5	3.8+	**yes**	vSub	none	3.5	3.6
	vSub_08	**L**	vSub	none	3.3	3.8	no	-	-	-	-
	vSub_09	**R**	vSub	none	3.6	3.9	no	-	-	-	-
	vSub_10	**R**	vSub	(weak vDG)	3.3	3.9	no	-	-	-	-
	vSub_11	**R**	vSub	none	3.3	3.8+	no	-	-	-	-
	vSub_12	**R**	vSub	none	3.8	3.9	**yes**	vSub	APir, AHipM,	3.4	3.9
	vSub_13	**R**	vSub	STr, MEnt	3.8	4	**yes**	vSub	STr, mEnt, APir, AHiPM	3.3	4
vCA1	vCA1_01	**L**	vCA1	vDG	3.3	3.7	no	-	vSub	-	-
	vCA1_02	**L**	vCA1	none	2.9	3.3	**yes**	vCA1	vDG	2.9	3.9
	vCA1_03	**L**	vCA1	none	3.2	3.5	no	-	-	-	-
	vCA1_04	**L**	vCA1	none	3.2	3.4	**yes**	vCA1	vDG	3.2	3.6
	vCA1_05	**L**	vCA1	none	3.4	3.5	no	-	-	-	-
	vCA1_06	**L**	vCA1	none	3.2	3.4	**yes**	vCA1	none	3.2	3.6
	vCA1_07	**L**	vCA1	vDG	2.9	3.6	**yes**	vCA1	vDG	3.3	3.5
	vCA1_08	**L**	vCA1	vDG	2.9	3.8	**yes**	vCA1	vDG	2.9	3.8
	vCA1_09	**L**	vCA1	vDG	2.9	3.9	**yes**	vCA1	vDG	2.9	3.9
	vCA1_10	**L**	vCA1	vDG	2.9	3.8	**yes**	vCA1	vDG	2.9	3.8
	vCA1_11	**R**	vCA1	vDG	3	3.9	**yes**	vCA1	vDG	3.4	3.9
	vCA1_12	**R**	vCA1	none	3.2	3.5	no	-	-	-	-
	vCA1_13	**R**	vCA1	vDG	2.9	3.9	**yes**	vCA1	vDG	2.9	3.9
	vCA1_14	**R**	vCA1	vDG, vCA3	2.9	3.9	**yes**	vCA1	vDG, vCA3	2.9	3.9
	vCA1_15	**R**	vCA1	none	3.4	3.6	**yes**	vCA1	vDG	3.5	3.8

Supplementary Table 1. Expression pattern in individual animals. Target- and off-target-expression regions and anterior-posterior (AP) extension of expression are shown for every mouse of the vSub and vCA1 groups. Same shown for the other hemisphere that was used in experiments with bilateral expression (right part of table). Numbers in the columns “AP-anterior” and “AP-posterior” show the approximate beginning and end, respectively, of the visible expression in mm posterior to bregma. 3.8+ indicates that the extension is likely beyond -3.8 mm, but not determined due to lack of posterior slices. AP-distances and regions were determined according to the atlas: Franklin & Paxinos, *The Mouse Brain in Stereotactic Coordinates*, 3rd ed., Academic Press, 2008. Abbreviations: APir, amygdalo-piriform transition area; AHipM, amygdalo-hippocampal posteromedial area; mEnt, medial entorhinal cortex; PMCo, posteromedial cortical amygdaloid nucleus; PLCo, posterolateral cortical amygdaloid nucleus; STr, transition area of the ventral subiculum; vCA3, CA3 subfield of the ventral hippocampus; vDG, dentate gyrus of the ventral hippocampus.

			Parametric analysis: one-way ANOVA				Non-parametric analysis: MWU-test					
			Effect of group		Dunnett-test, one-sided		vSub			vCA1		
Assay	Variable	Figure	F	p	vSub	vCA1	U	Z	p	U	Z	p
Locomotor activity, 20 Hz	Distance during stim	1d	9.5	0.000	0.086	0.000	80	-2.0	0.045	52	-3.4	0.001
	Distance post-stim	1d	6.6	0.003	0.520	0.001	121	-0.5	0.583	86	-2.3	0.022
	Relative dist. during stim	1f	6.4	0.003	0.046	0.001	51	-3.0	0.002	50	-3.4	0.001
	Relative dist. post-stim	1f	6.0	0.005	0.631	0.002	133	-0.1	0.901	98	-1.9	0.056
Locomotor activity, 5 Hz	Distance during stim	1e	1.6	0.21858			110	-0.5	0.604	68	-2.1	0.033
	Distance post-stim	1e	0.1	0.86173			112	-0.4	0.659	122	-0.1	0.954
	Relative dist. during stim	1g	1.3	0.2707			111	-0.5	0.631	94	-1.1	0.258
	Relative dist. post-stim	1g	0.9	0.40919			113	-0.4	0.687	102	-0.8	0.409
Hole-board, 20 Hz	Poke-holes during stim	2b	2.1	0.137	0.052	0.174	69.5	-2.4	0.018	104	-1.7	0.086
EPM, 20 Hz	Distance moved	3b	0.5	0.615			131	-0.2	0.845	98	-1.0	0.295
	Entries to open arm	3c	1.3	0.290			115	-0.8	0.443	109	-0.6	0.519
	Time in open arm	3d	1.6	0.218			108	-1.0	0.312	118.5	-0.3	0.779
	Preference for open arm	3e	1.6	0.211			104	-1.2	0.244	116.5	-0.4	0.718
Y-maze, SP-stimulation, 5Hz	Distance, SP	4b	3.4	0.041	0.018	0.065	76	-2.1	0.032	94	-2.0	0.042
	Distance, TP	4c	0.7	0.513			115	-0.8	0.446	139	-0.6	0.553
	SNP, entries	4e	0.1	0.906			128	-0.3	0.762	146	-0.4	0.711
	SNP, time	4f	1.3	0.289			115	-0.8	0.446	134	-0.8	0.451
	Time in novel arm	4d	1.3	0.278			111	-0.9	0.366	140	-0.6	0.574
	Time in familiar arm	4d	0.9	0.421			127	-0.3	0.736	126	-1.0	0.312
Y-maze, SP-stimulation, 20Hz	Distance, SP	4b	0.6	0.539			127	-0.3	0.736	145	-0.4	0.688
	Distance, TP	4c	2.3	0.109	0.858	0.083	120	-0.6	0.559	133	-0.8	0.432
	SNP, entries	4e	4.1	0.023	0.378	0.007	122	-0.5	0.605	69.5	-2.6	0.009
	SNP, time	4f	3.0	0.058	0.536	0.021	118	-0.7	0.512	83	-2.2	0.031
	Time in novel arm	4d	2.4	0.101	0.690	0.046	132	-0.2	0.873	89	-2.0	0.051
	Time in familiar arm	4d	1.9	0.168	0.562	0.063	119	-0.6	0.535	88.5	-2.0	0.049
Y-maze, TP-stimulation, 20Hz	Distance, SP	4b	0.3	0.757			125	-0.4	0.684	140	-0.6	0.574
	Distance, TP	4c	8.0	0.001	0.229	0.000	107	-1.0	0.296	69	-2.8	0.005
	SNP, entries	4e	0.8	0.468			105	-1.1	0.262	153.5	-0.1	0.897
	SNP, time	4f	0.4	0.661			117	-0.7	0.490	153	-0.1	0.885
	Time in novel arm	4d	0.3	0.774			115	-0.8	0.446	140.5	-0.5	0.585
	Time in familiar arm	4d	0.6	0.535			117.5	-0.7	0.501	135	-0.7	0.470
NOR, SP-stimulation	SP interaction number	5b	0.9	0.433			92	-1.4	0.161	100	-0.8	0.436
	SP interaction time	5c	0.6	0.528			93	-1.4	0.173	105	-0.6	0.559
	TP interaction number, novel	5d	1.5	0.239			119	-0.4	0.684	83	-1.4	0.149
	TP interaction number, familiar	5d	2.7	0.082	0.866	0.065	122.5	-0.3	0.782	78.5	-1.6	0.105
	TP interaction time, novel	5e	1.7	0.190	0.392	0.065	107	-0.8	0.397	68.5	-2.0	0.045
	TP interaction time, familiar	5e	2.2	0.121	0.826	0.079	125.5	-0.2	0.868	85	-1.4	0.173
	TP preference, number	5f	0.6	0.560			103	-1.0	0.319	108	-0.5	0.640
	TP preference, time	5g	0.1	0.897			125	-0.2	0.854	107	-0.5	0.613
NOR, TP-stimulation	SP interaction number	5b	1.8	0.174	0.131	0.872	84.5	-1.7	0.093	106.5	-0.1	0.885
	SP interaction time	5c	3.8	0.031	0.013	0.651	68	-2.3	0.022	109	0.0	0.967
	TP interaction number, novel	5d	0.5	0.639			121.5	-0.3	0.753	100.5	-0.4	0.694
	TP interaction number, familiar	5d	6.9	0.003	0.116	0.001	73.5	-2.1	0.037	45.5	-2.7	0.008
	TP interaction time, novel	5e	1.4	0.264			103.5	-1.0	0.329	105.5	-0.2	0.853
	TP interaction time, familiar	5e	5.3	0.009	0.030	0.004	69.5	-2.2	0.026	54	-2.3	0.021
	TP preference, number	5f	5.6	0.007	0.153	0.002	107	-0.8	0.396	34	-3.1	0.002
	TP preference, time	5g	6.1	0.005	0.198	0.001	104	-1.0	0.337	38	-3.0	0.003
TM, 20Hz unilat., SP	%correct	6b	0.6	0.541			74.5	-0.4	0.667	87.5	-0.1	0.899
TM, 20Hz unilat., CP	%correct	6b	0.6	0.534			59	-1.3	0.189	62	-1.5	0.140
TM, 5Hz unilat., SP	%correct	6c	0.2	0.813			21	-0.5	0.595	30.5	-0.5	0.607
TM, 5Hz unilat., CP	%correct	6c	0.5	0.611			19.5	-0.7	0.464	35	0.0	1.000
TM, 20Hz bilat., SP	%correct	6d	1.5	0.246			18.5	-1.3	0.205	48.5	-0.8	0.441
TM, 20Hz bilat., CP	%correct	6d	2.8	0.079	0.326	0.024	24	-0.6	0.517	24.5	-2.4	0.018
TM, 20Hz bilat., SP, occluded	%correct	6e	6.8	0.004	0.837	0.003	29	-0.6	0.562	30.5	-2.3	0.019
TM, 20Hz bilat., CP, occluded	%correct	6e	1.5	0.248			15.5	-1.9	0.064	61	-0.5	0.585
TM, 20Hz unilat., SP, occluded	%correct	6f	1.0	0.378			12	-0.6	0.565	14	-0.6	0.552
TM, 20Hz unilat., CP, occluded	%correct	6f	5.4	0.017	0.276	0.006	9	-1.2	0.227	2.5	-2.5	0.011
Social interaction	IA number, stim 1	7b	0.0	0.972			94.5	-0.3	0.739	107	-0.1	0.883
	IA number, post-stim 1	7b	3.7	0.035	0.774	0.024	100	-0.1	0.929	58.5	-2.2	0.029
	IA number, stim 2	7b	0.2	0.829			73.5	-0.5	0.638	52	-1.3	0.199
	IA number, post-stim 2	7b	0.4	0.657			81.5	-0.1	0.958	58	-0.9	0.342
	IA time, stim 1	7c	0.8	0.471			92.5	-0.4	0.674	109	-0.1	0.950
	IA time, post-stim 1	7c	0.3	0.768			92.5	-0.4	0.674	94	-0.7	0.490
	IA time, stim 2	7c	0.9	0.426			55	-1.4	0.153	67.5	-0.4	0.677
	IA time, post-stim 2	7c	0.5	0.596			75.5	-0.4	0.716	57.5	-1.0	0.331

Supplementary Table S2. Parametric and non-parametric between-subject analysis. Results (test statistic and exact *p*-values) of between-subject comparisons with one-way ANOVA and one-sided Dunnett's post-hoc test (left, parametric) and with MWU-test (right,

non-parametric) for all important behavioural variables shown in the main figures (Fig. 1-7) of the manuscript. *Red* and *blue* indicate that the Dunnett's test result reflects differences whereby values from the experimental groups are *larger* or *smaller*, respectively, than those of the control group, as based on the hypothesis resulting from the actual data distribution. Strong and weak green reflect significance ($p < 0.05$) and trends ($p < 0.2$ for ANOVA, $p < 0.1$ for MWU-test), respectively. $p < 0.05$ is also indicated by bold font for all tests.

RM-ANOVA across groups; within-subject variable: novelty of stimulus		Figure	group		novelty		interaction		Sidak post-hoc test (p), within-subject			Sidak post-hoc test (p), vCA1 vs. Ctrl	
			F	p	F	p	F	p	Ctrl	vSub	vCA1	Novel	Familiar
Y-maze, SP-stimulation, 5Hz	Time in arms	4d	0.6	0.556	23.2	**0.000**	1.1	0.329	**0.001**	**0.001**	0.129	0.872	0.586
Y-maze, SP-stimulation, 20Hz	Time in arms	4d	0.4	0.660	165.2	**0.000**	2.8	0.075	**0.000**	**0.000**	**0.000**	0.142	0.191
NOR, SP-stimulation	Time with objects	5e	2.1	0.132	66.0	**0.000**	0.9	0.417	**0.000**	**0.000**	**0.000**	0.196	0.238
	Interactions with objects	5d	2.2	0.128	48.5	**0.000**	0.8	0.436	**0.000**	**0.000**	**0.002**	0.259	0.198
NOR, TP-stimulation	Time with objects	5e	2.0	0.148	42.4	**0.000**	3.1	0.054	**0.000**	**0.000**	0.163	0.999	**0.013**
	Interactions with objects	5d	2.6	0.090	30.1	**0.000**	3.5	**0.041**	**0.000**	**0.000**	0.402	0.876	**0.002**

Supplementary Table S3. Parametric analysis of effects of novelty with repeated-measures two-way ANOVA. Results of repeated-measures ANOVA comparing novel and familiar stimuli (within-subject factor) and groups (between-subject factor). *P*-values of within-subject and – for vCA1 vs. Ctrl – between-subject post-hoc comparisons with two-sided Sidak-test are indicated on the right. Strong and weak green reflect p < 0.05 and p < 0.1, respectively.

RM-ANOVA across groups; within-subject variable: stimulation		Within-subject variable	group		stimulation		interaction		group differences within stim-condition			effect of stimulation within group			within-subject comparison within vCA1		
			F	p	F	p	F	p	SP	TP/CP	BL	Ctrl	vSub	vCA1	SP vs TP/CP	BL vs SP	BL vs CP
Y-maze, SP-stimulation	Preference (SNP, time)	5 vs 20 Hz	2.0	0.141	3.1	0.087	0.3	0.747	-	-	-	-	-	-	-	-	-
	Preference (SNP, visits)	5 vs 20 Hz	1.8	0.180	0.2	0.665	0.7	0.499	-	-	-	-	-	-	-	-	-
NOR	Preference (time)	SP vs TP	4.5	0.017	2.0	0.162	1.8	0.182	0.914	0.005	-	0.655	0.571	0.051	0.051	-	-
	Preference (interactions)	SP vs TP	5.3	0.009	1.2	0.281	1.5	0.236	0.502	0.007	-	0.437	0.336	0.175	0.175	-	-
T-maze, 20Hz unilateral	%correct	BL vs SP vs TP	1.1	0.336	1.6	0.203	0.4	0.803	-	-	-	-	-	-	-	-	-
T-maze, 5Hz unilateral	%correct	BL vs SP vs TP	0.3	0.713	0.2	0.856	0.5	0.719	-	-	-	-	-	-	-	-	-
T-maze, 20Hz bilateral	%correct	BL vs SP vs TP	2.3	0.118	6.6	0.003	1.9	0.127	-	-	-	0.002	0.208	0.230	0.333	0.066	0.004
T-maze, 20Hz bilateral, occluded	%correct	BL vs SP vs TP	2.1	0.141	4.7	0.013	4.8	0.002	0.004	0.248	0.653	0.404	0.083	0.000	0.043	0.000	0.182
T-maze, 20Hz unilateral, occluded	%correct	BL vs SP vs TP	2.5	0.114	4.9	0.015	3.5	0.018	0.378	0.017	0.524	0.837	0.746	0.000	0.007	0.120	0.001

Supplementary Table S4. Parametric analysis of effects of stimulation with repeated-measures two-way ANOVA. Results of repeated-measures ANOVA comparing stimulation phase or frequency (within-subject factor, as indicated) and groups (between-subject factor). *P*-values of within-subject and between-subject post-hoc comparisons with two-sided Sidak-test are indicated on the right, where applicable due to significant main effects. Strong and weak green reflect p < 0.05 and p < 0.1, respectively.

Supplementary Figures

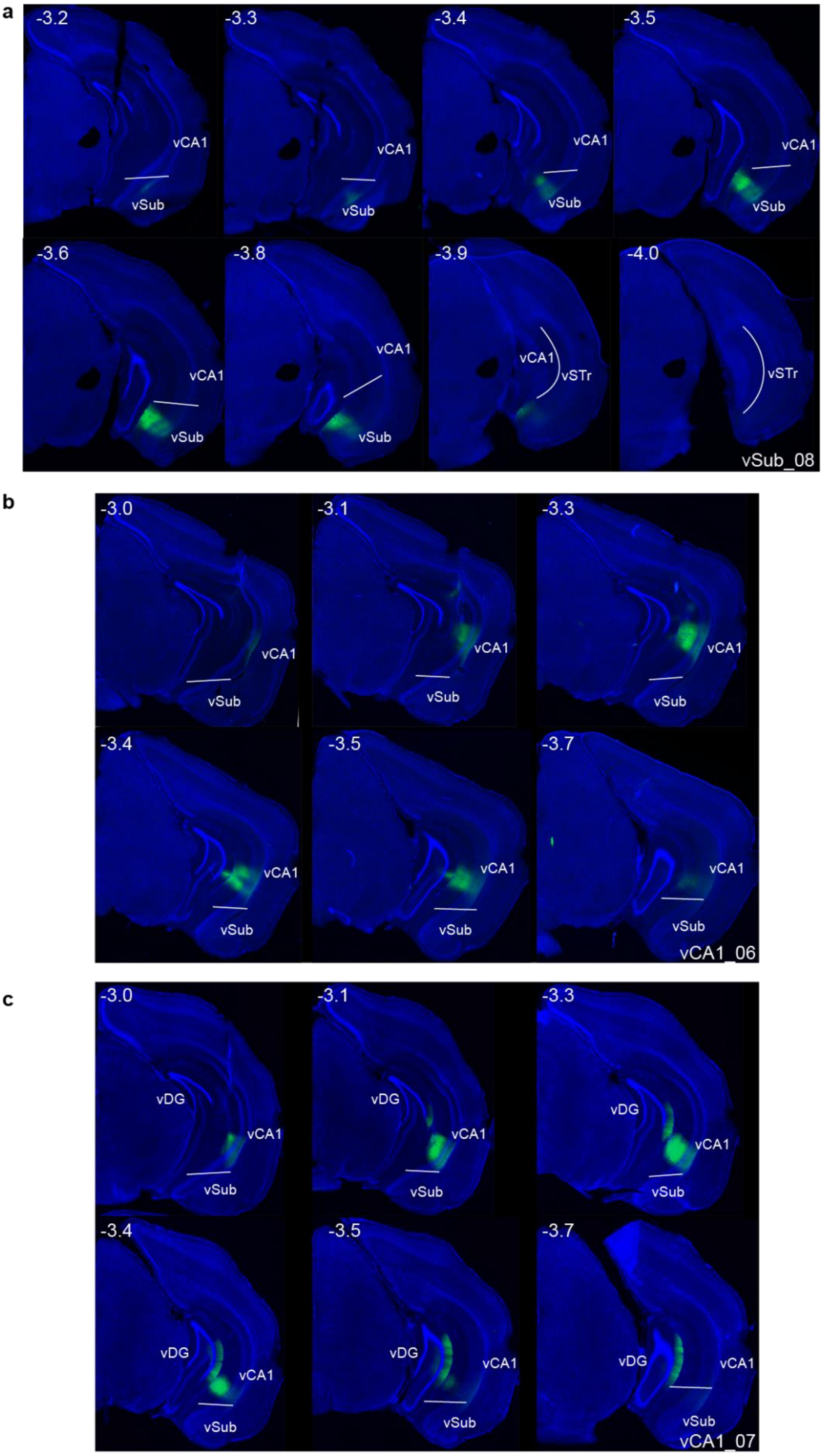

Supplementary Figure 1. Chronos-expression patterns. Coronal slices of left hemispheres showing expression of Chronos-eGFP (green) in anterior-posterior order (from left to right and

top to bottom) for animal from the vSub-group (**a**), for an animal from the vCA1-group *without* off-target expression (**b**), and for an animal from the vCA1-group *with* strong off-target expression in the lateral molecular layer of the ventral dentate gyrus (vDG; **c**). DAPI shown in blue. AP-distance relative to bregma shown at the top-left of each subpanel. Border between ventral subiculum (vSub) and ventral CA1 (vCA1) subfields indicated by white line. STr, transition area of the ventral subiculum. Animal-IDs (referring to Supplementary Table 1) indicated in the bottom-right corner. AP-distances and regions were determined according to the atlas: Franklin & Paxinos, *The Mouse Brain in Stereotactic Coordinates*, 3rd ed., Academic Press, 2008

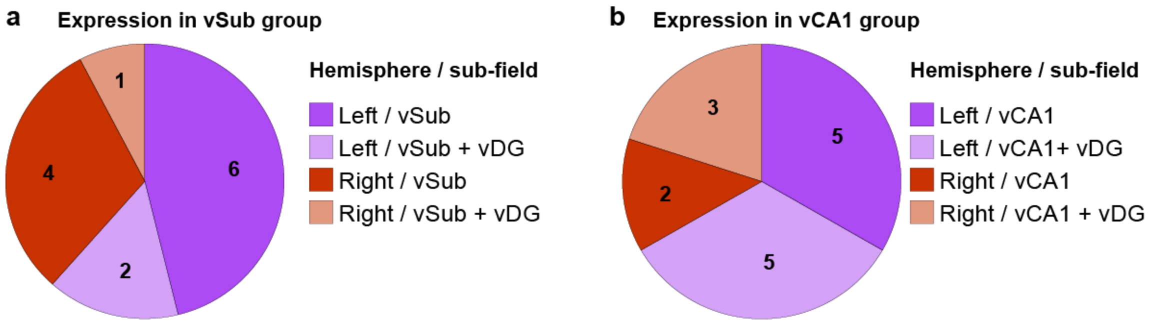

Supplementary Figure 2. Stimulated hemisphere and off-target expression. Number of mice (bold numbers) from the vSub (**a**) and vCA1 (**b**) groups in which the left or right hemisphere was used for stimulation and which showed absence or presence of off-target expression in ventral dentate gyrus (vDG), as indicated in the colour legend.

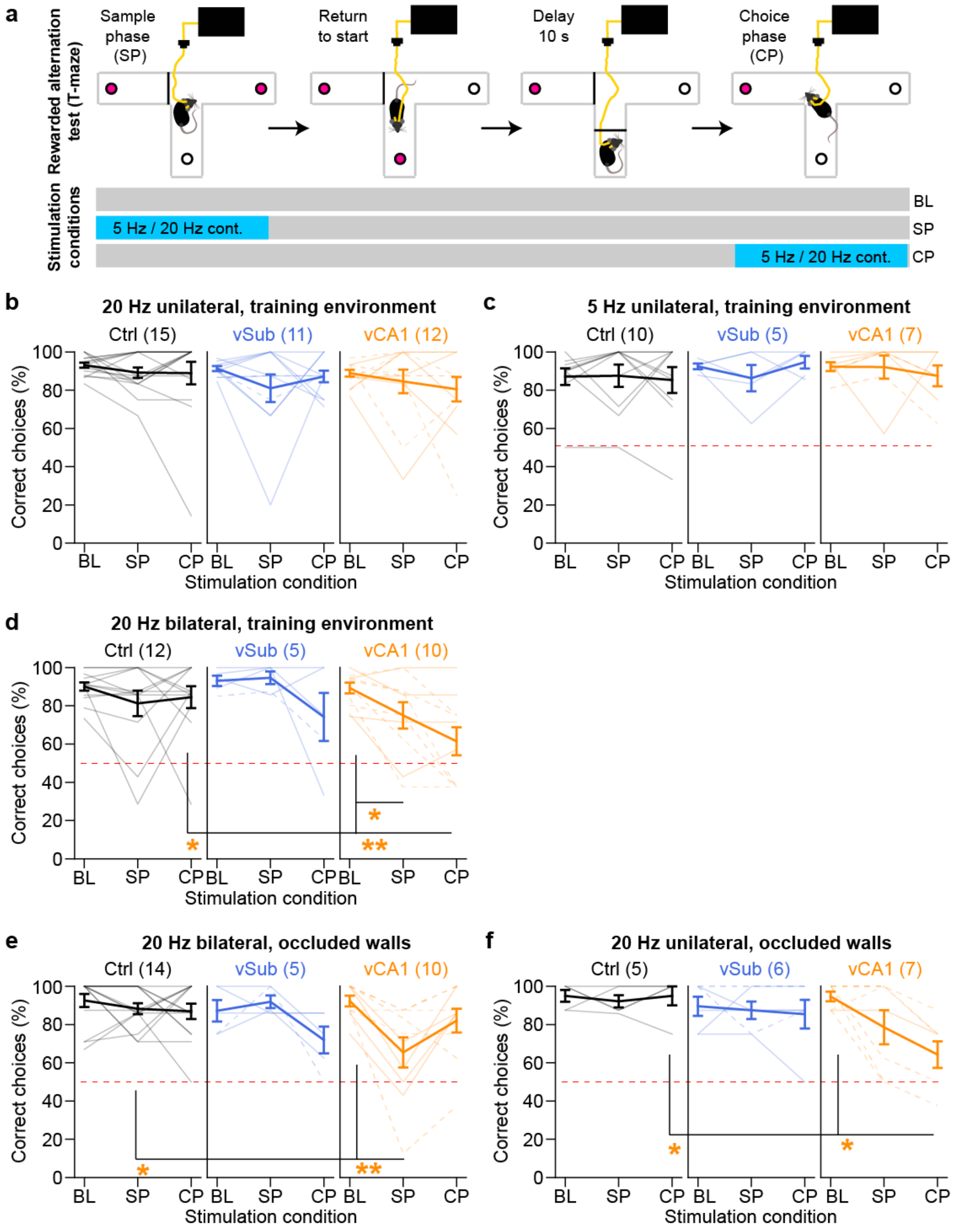

Supplementary Fig. 3. Impairment of spatial working memory by optogenetic stimulation of vCA1. Same data as Fig. 6 but with an indication of animals with vDG off-target expression by dashed lines. (**a**) Time-course of a single trial of the T-maze rewarded alternation test, whereby optogenetic stimulation was either omitted (baseline, BL) or delivered continuously at 5 or 20 Hz during the SP or the CP. (**b-f**) Working memory accuracy, expressed as %correct choices for distinct stimulation conditions: unilateral stimulation with 20 Hz (b) or 5 Hz (c), or bilateral stimulation with 20 Hz (d) in the familiar environment in which the animals have received their alternation training sessions; 20 Hz bilateral (e) or unilateral (f) stimulation with occluded side-walls. *N*-numbers (stated in brackets above each sub-panel) in (c) and (f) are reduced because only batch 1 (c) or batch 2 (f) of the cohort conducted these experiments; *N*-number in (d) and (e) are reduced because only animals with bilateral

expression were included in the analysis of bilateral stimulation experiments. Further reductions of N-numbers, compared to the original cohort, are due to insufficient participation in the task or lack of consistent reward consumption. Individual lines represent individual subjects, error bars represent s.e.m.; red dashed line indicates chance level; asterisks within each sub-panel indicate significant difference in pairwise within-subject comparisons of either one of the stimulation conditions compared to the baseline condition (Wilcoxon signed-ranks test); asterisks in the Ctrl subpanels indicate non-parametric paired between-subject comparisons to the control group (Ctrl, black; MWU-test). * $p < 0.05$; ** $p < 0.01$.
